# The conserved histone chaperone LIN‐53 is required for normal lifespan and maintenance of muscle integrity in *Caenorhabditis elegans*

**DOI:** 10.1111/acel.13012

**Published:** 2019-08-09

**Authors:** Stefanie Müthel, Bora Uyar, Mei He, Anne Krause, Burcu Vitrinel, Selman Bulut, Djordje Vasiljevic, Iris Marchal, Stefan Kempa, Altuna Akalin, Baris Tursun

**Affiliations:** ^1^ Berlin Institute of Medical Systems Biology Berlin Germany; ^2^ Max Delbrück Center for Molecular Medicine in the Helmholtz Association Berlin Germany; ^3^ Present address: Muscle Research Unit, Experimental and Clinical Research Center (ECRC) of Charité, Max Delbrück Center for Molecular Medicine Universitätsmedizin Berlin Berlin Germany; ^4^ Present address: Department of Biology, Center for Developmental Genetics New York University New York NY USA; ^5^ Present address: College of Life Science Northeast Forestry University Harbin China

**Keywords:** aging, *Caenorhabditis elegans*, chromatin, epigenetics, healthspan, metabolome

## Abstract

Whether extension of lifespan provides an extended time without health deteriorations is an important issue for human aging. However, to which degree lifespan and aspects of healthspan regulation might be linked is not well understood. Chromatin factors could be involved in linking both aging aspects, as epigenetic mechanisms bridge regulation of different biological processes. The epigenetic factor LIN‐53 (RBBP4/7) associates with different chromatin‐regulating complexes to safeguard cell identities in *Caenorhabditis elegans* as well as mammals, and has a role in preventing memory loss and premature aging in humans. We show that LIN‐53 interacts with the nucleosome remodeling and deacetylase (NuRD) complex in *C. elegans* muscles to ensure functional muscles during postembryonic development and in adults. While mutants for other NuRD members show a normal lifespan, animals lacking LIN‐53 die early because LIN‐53 depletion affects also the histone deacetylase complex Sin3, which is required for a normal lifespan. To determine why *lin‐53* and *sin‐3* mutants die early, we performed transcriptome and metabolomic analysis revealing that levels of the disaccharide trehalose are significantly decreased in both mutants. As trehalose is required for normal lifespan in *C. elegans, lin‐53* and *sin‐3* mutants could be rescued by either feeding with trehalose or increasing trehalose levels via the insulin/IGF1 signaling pathway. Overall, our findings suggest that LIN‐53 is required for maintaining lifespan and muscle integrity through discrete chromatin regulatory mechanisms. Since both LIN‐53 and its mammalian homologs safeguard cell identities, it is conceivable that its implication in lifespan regulation is also evolutionarily conserved.

## 1 INTRODUCTION

The decline of physical condition and the onset of diseases such as cancer, diabetes, or dementia are important issues during aging. Age‐associated deterioration of health has gained importance as the human life expectancy constantly increases worldwide. It has been predicted that in 2050, adults over the age of 80 will triple compared with the year 2015 (Jaul & Barron, 2017). Hence, an important aspect of aging is whether increasing lifespan would also extend the healthspan, meaning the time of life without unfavorable health conditions. However, genetic factors that play a role in linking healthspan with lifespan regulation are largely unknown. Aging regulation by chromatin‐regulating factors could play a role in linking lifespan with healthspan as loss of epigenetic gene regulation diminishes cell fate safeguarding (Kolundzic et al., 2018; Onder et al., 2012; Yadav, Quivy, & Almouzni, 2018), declines stem cell health (Brunet & Rando, 2017; Ren, Ocampo, Liu, & Belmonte, 2017), impairs muscle regeneration (Guasconi & Puri, 2009), and shortens lifespan of organisms (Field & Adams, 2017; Greer et al., 2010). Prominent examples include conserved NAD(+)‐dependent deacetylating enzymes known as sirtuins (e.g., SIR‐2.1 in *C. elegans*), which were originally identified in yeast as histone H3 and histone H4 deacetylases in order to silence gene expression (Braunstein, Sobel, Allis, Turner, & Broach, 1996). Besides histones also other proteins such transcription factors are targeted by sirtuins (reviewed by Chang and Guarente (2014) and Houtkooper, Pirinen, and Auwerx (2012)). For instance, overexpression of *sir‐2.1* in *C. elegans* causes lifespan extension through modulating the activity of the critical lifespan‐regulating FOXO transcription factor DAF‐16 (Tissenbaum & Guarente, 2001) (Berdichevsky, Viswanathan, Horvitz, & Guarente, 2006). Consequently, acetyltransferases such as the *C. elegans* CBP/p300 homolog CBP‐1 also affect lifespan and metabolic pathways by acetylating DAF‐16 (reviewed in Tissenbaum (2018) and Denzel, Lapierre, and Mack (2019)). Conversely, the feedback of metabolites on epigenetic regulation is increasingly apparent and therefore emerging as a critical layer of gene regulation not only for aging regulation but also for cellular plasticity (review in Cliff and Dalton (2017) and Sperber et al. (2015)).

One specific type of epigenetic regulators is histone chaperones, which are proteins that directly interact with histones and function as a scaffold for chromatin‐modifying protein complexes (Hammond, Strømme, Huang, Patel, & Groth, 2017). They are important for folding, oligomerization, post‐translational modifications, nucleosome assembly, and genomic location of histones (Hammond et al., 2017). The *C. elegans* histone chaperone LIN‐53 is highly conserved and known as RBBP4/7 (also as CAF‐1p48) in mammals. LIN‐53 and its homologs can be found in different protein complexes that regulate the repressive and active state of chromatin (Lu & Horvitz, 1998) (Loyola & Almouzni, 2004) (Eitoku, Sato, Senda, & Horikoshi, 2008). Among those complexes are PRC2 (polycomb repressive complex 2 (Margueron & Reinberg, 2011)), Sin3 histone deacetylase complex (Sin3 HDAC) (Nicolas et al., 2000), NuRD (nucleosome remodeling and deacetylase complex; Allen, Wade, & Kutateladze, 2013), CAF‐1 histone chaperone complex (Verreault, Kaufman, Kobayashi, & Stillman, 1996), and DRM (Dp/Rb/Muv; Harrison, Ceol, Lu, & Horvitz, 2006). In *C. elegans,* LIN‐53 was shown to interact with the Rb homolog LIN‐35 to antagonize the Ras signaling pathway (Lu & Horvitz, 1998). Moreover, LIN‐53 and its mammalian homolog RBBP4/7 safeguard cells against reprogramming (Cheloufi et al., 2015; Tursun, Patel, Kratsios, & Hobert, 2011) and have been implicated in age‐related memory loss and premature aging in humans (Pavlopoulos et al., 2013; Pegoraro et al., 2009).

In this study, we revealed that LIN‐53 is required for ensuring muscle integrity starting at the last larval stage to maintain healthy motility and normal lifespan in *C. elegans*. Notably, the muscle defects and short lifespan caused by loss of *lin‐53* can be unlinked based on different chromatin‐regulating complexes. LIN‐53 is interacting with the NuRD complex to maintain muscle integrity and proper motility but requires the Sin3 complex to ensure normal lifespan. To understand why *lin‐53* and *sin‐3* mutants have a shortened lifespan, we analyzed the transcriptome as well as metabolome of mutant animals. Loss of LIN‐53 or SIN‐3 leads to a strong decrease in trehalose levels — a disaccharide that is required for normal lifespan (Honda, Tanaka, & Honda, 2010; Seo, Kingsley, Walker, Mondoux, & Tissenbaum, 2018). Restoring trehalose levels by feeding, or genetically via the Insulin/IGF1 signaling (IIS) pathway, suppressed the short lifespan of *lin‐53* and *sin‐3* mutants, supporting the idea that LIN‐53 and SIN‐3 are required to maintain a normal lifespan via ensuring the homeostasis of metabolites such as trehalose.

Overall, our findings suggest that the epigenetic factor LIN‐53 links muscle development and maintenance with lifespan regulation in *C. elegans*. As LIN‐53 is a highly conserved chromatin regulator with an evolutionarily conserved role in cell fate safeguarding (Cheloufi et al., 2015; Cheloufi & Hochedlinger, 2017; Tursun et al., 2011), it is conceivable that its homologs regulate lifespan and healthspan aspects in other species. Hence, our findings provide an initial framework for elucidating how lifespan and healthspan regulation might be linked through epigenetic factors, which could be of high relevance for human health and aging.

## 2 RESULTS

### 2.1 Loss of LIN‐53 results in muscle and locomotion defects

**Figure 1:**
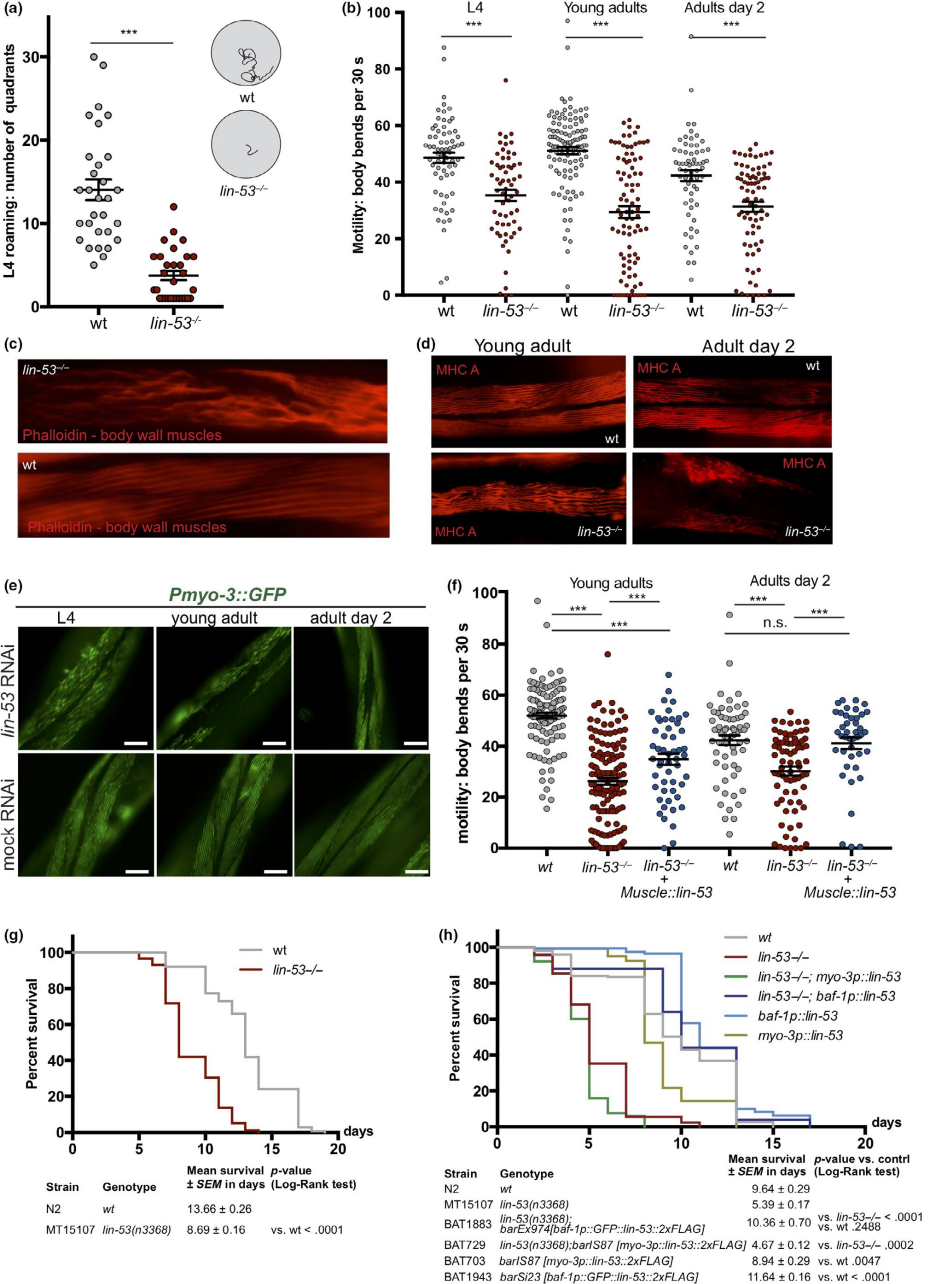
Loss of LIN‐53 causes locomotion defects and short lifespan. (a) L4 control and *lin‐53* mutant worms were put on solid agar plates, and the movement on solid agar was monitored. *lin‐53* mutant animals move significantly slower than control animals. Statistical analysis was carried out using unpaired *t*‐test; ***p < .0001. (b) Control and *lin‐53* mutant animals at three different developmental stages (L4 larvae, young adult animals, and adults at day 2) were put in M9 medium, motility was recorded, and the body bends were counted using the ImageJ wrMTrck plugin. At all developmental stages, *lin‐53* mutants swim significantly slower than control animals. Statistical analysis was carried out using one‐way ANOVA; ***p < .0001. (c) Phalloidin binds to F‐actin in body wall muscles. In *lin‐53* mutant animals, the muscle structure is disrupted compared with control animals. (d) The muscle structure of *lin‐53* mutants and control animals at two different developmental stages (young adult and adult day 2) was analyzed using an antibody against MHC in immunostaining. The muscle structure is disrupted in *lin‐53* mutants compared with control animals at both stages. (e) Worms expressing a *Pmyo‐3::GFP* reporter were subjected to control and *lin‐53* RNAi at different developmental stages (L4, young adults, adult day 2). At all three stages, a muscle structure disruption is detectable upon loss of *lin‐53.* (f) Expression of recombinant LIN‐53 only in muscles rescues the motility defect of *lin‐53* mutants. Statistical analysis was carried out using one‐way ANOVA; ***p < .0001, ns = not significant. (g) Depletion of *lin‐53* decreases the lifespan of *C. elegans* by 5 days (*p*‐value < .0001). wt animals (gray line; mean lifespan 13.66 ± 0.26 days) and *lin‐53* mutants (red line; mean lifespan 8.69 ± 0.16 days). Triplicate experiments with 40 animals per repeat. Survival analysis was carried using Kaplan–Meier estimator, and *p*‐value was calculated using log‐rank test. (h) The short lifespan of *lin‐53* mutants is not rescued upon overexpression of *lin‐53* in muscles. Upon ubiquitous *lin‐53* expression using the *baf‐1* promoter, lifespan of *lin‐53* mutants is rescued and wt *lin‐53* animals live 2 days longer than control animals. For the lifespan assay, animals were kept continuously at 25°C. To distinguish *lin‐53(n3368)* mutants animals from wt animals, the “protruding vulva” (*pvul*) phenotype was used as a marker for efficient *lin‐53* RNAi. Upon occurrence of pvul, the day count for lifespan scoring started (at L4 stage). Triplicate experiments with 40 animals per repeat. Survival analysis was done using Kaplan–Meier estimator, and *p*‐value was calculated using log‐rank test

The role of the highly conserved histone chaperone LIN‐53 in somatic tissues of *C. elegans* is poorly understood. We therefore examined *lin‐53* null mutants and noticed a severe movement defect for *lin‐53(n3368)* animals. Starting postembryonically at  the last larval L4 stage, they exhibit decreased mobility on solid agar plates (Figure 1a) as well as in liquid when compared to wild‐type animals (Figure 1b). Such motility defects can point to an impaired muscle apparatus, which prompted us to stain muscles and assess their integrity in *lin‐53* mutants. Using fluorescent phalloidin, which binds to F‐actin fibers in muscles (Figure 1c), and an antibody against the myosin heavy chain (MHC) component of body wall muscles (Figure 1d), we observed disrupted muscle structures in *lin‐53* mutants (Figure 1c and d). The decline in muscle integrity upon *lin‐53* depletion is also evident based on animals expressing a *Pmyo‐3::GFP* reporter (Figure 1e). These muscle phenotypes in *lin‐53* mutants are cell‐autonomous effects as muscle‐specific RNAi against *lin‐53,* by using a hairpin construct (*myo‐3p::lin‐53_*HP), also leads to muscle and motility defects (Figure S1A and B). Consequently, the muscle and motility defects in *lin‐53* mutants can be rescued by expressing full‐length LIN‐53 specifically in muscles using the *myo‐3* promoter (Figures 1f and S1C). Interestingly, wt animals overexpressing *myo‐3p::lin‐53* or *myo‐3p::GFP::lin‐53* move significantly better than control animals, suggesting that the overexpression of LIN‐53 in muscles has a beneficial effect to maintain the motility in adult and aged animals (Figure S1D).

Overall, our findings suggest that LIN‐53 is required to establish and maintain muscle integrity in order to prevent the decline of locomotion capabilities in *C. elegans.*

### 2.2 Loss of LIN‐53 shortens lifespan

Since deterioration of coordinated movement is associated with aging (Herndon et al., 2002), we wondered whether *lin‐53* mutants suffer from a short lifespan. Lifespan assays revealed that *lin‐53(n3368)* mutants have an average lifespan which is around 40% shorter than wt animals (Figure 1g and Table S1). A shortened lifespan is also evident in animals carrying the CRISPR/Cas9‐generated *lin‐53* null allele *(bar19)* (Figure S1E and Table S1). Interestingly, while the short lifespan of *lin‐53* mutants is not rescued upon overexpression of *lin‐53* in muscles (*myo‐3p::lin‐53*) it rescues the motility defect as shown earlier (Figure 1f and Figure S1D). In contrast, ubiquitous expression of recombinant LIN‐53 using the *baf‐1* promoter (*baf‐1p::lin‐53*; Figure 1h) rescues the short lifespan in *lin‐53* mutants confirming the functionality of heterologously expressed LIN‐53 fusion proteins. Moreover, *baf‐1p::lin‐53* animals without *lin‐53* mutation in the background show an extended lifespan by approximately 2–3 days upon ubiquitous *lin‐53* expression compared with control wt animals (Figures 1h and S1E).

Our findings show that *lin‐53* is required to establish and maintain healthy locomotion and a normal lifespan, raising the possibility that *lin‐53* links lifespan with regulation of muscle development and healthspan aspects by separating survival from muscle atrophy.

### 2.3 The muscle defect of *lin‐53* mutants is phenocopied upon loss of the NuRD complex

**Figure 2:**
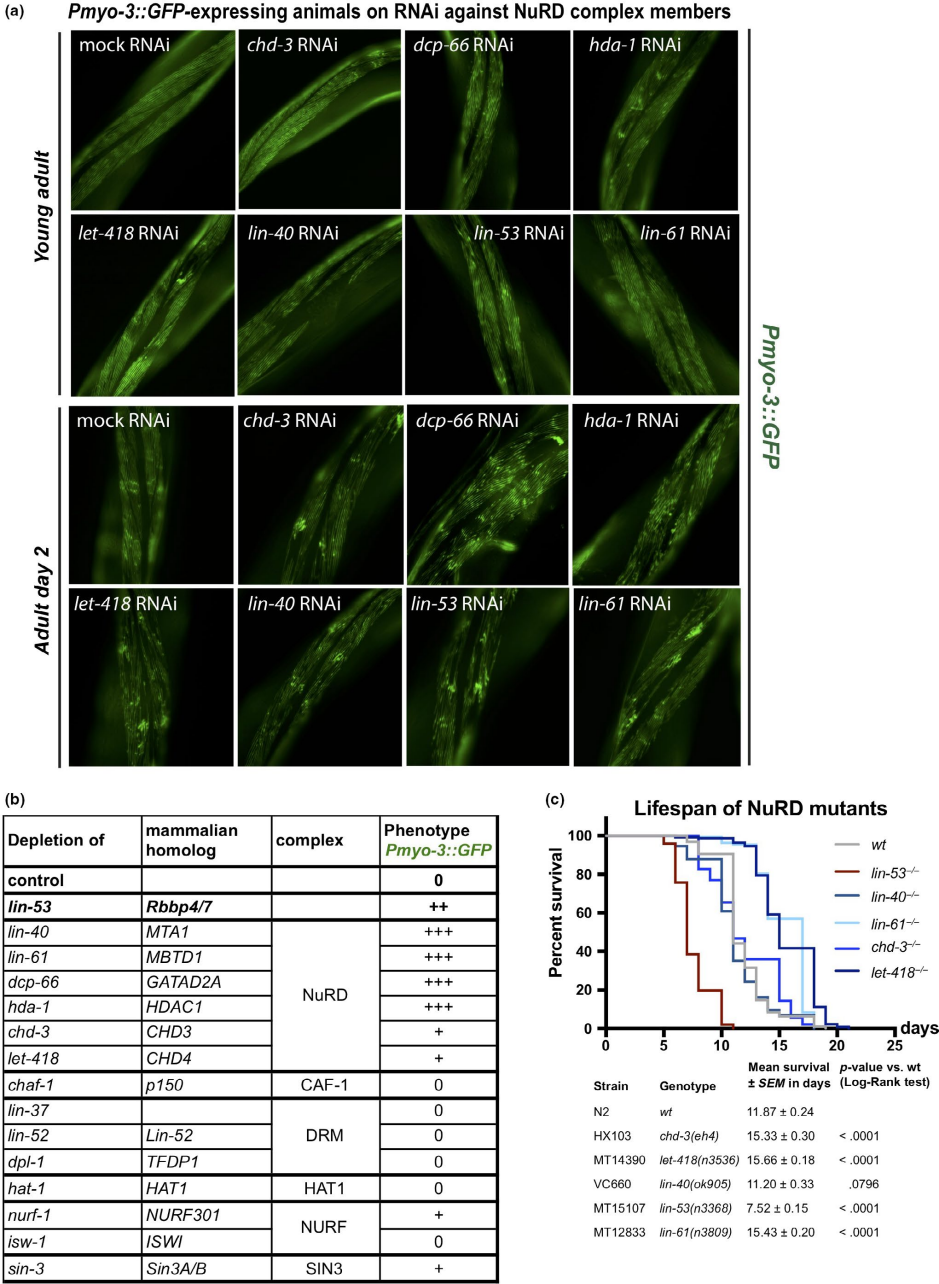
Loss of NuRD complex members phenocopy muscle defects of *lin‐53* mutants. (a) Screening whether RNAi against genes encoding LIN‐53‐interacting partners phenocopies muscle integrity disruption as seen in *lin‐53* mutants based on *Pmyo‐3::GFP*. Representative fluorescent pictures of the *Pmyo‐3::GFP* reporter are shown. (b) RNAi‐depletion of NuRD complex members phenocopies *lin‐53^−/−^* muscle phenotype. Disruption of the muscle structure was scored as follows: 0 = no effect, + = slight effect, ++ = medium defect, and +++ = strong defect. (c) Depletion of NuRD complex members does not phenocopy the short lifespan of *lin‐53* mutants. Lifespan assay was carried out continuously at 25°C and the survival analysis was done using Kaplan–Meier estimator, and *p*‐value was calculated using log‐rank test. The experiment was done at least two times with at least 40 animals per repeat

LIN‐53 is part of several different chromatin‐regulating complexes including the PRC2, NuRD, Sin3, and DRM complexes (Eitoku et al., 2008; Loyola & Almouzni, 2004; Lu & Horvitz, 1998). Hence, we wondered whether the observed phenotypes in *lin‐53*‐depleted animals are due to the altered function of a distinct LIN‐53‐containing complex. We generated an RNAi sublibrary targeting all known LIN‐53 interaction partners and tested whether depletion of any of the interaction partners phenocopies the observed muscle defects based on the *Pmyo‐3::GFP* reporter (Figure 2a and b, Figure S2F–H). An impairment of muscle integrity became evident upon knockdown of genes encoding for NuRD complex members such as *lin‐61*, *lin‐40*, *dcp‐66,* and, to a lesser degree, *let‐418* and its paralog *chd‐3* (Figure 2a and b). This observation was further confirmed by immunostaining of myosin (MHC) in mutant animals for NuRD complex members (Figure S2A), and motility assays (Figure S2B). Since combination of *lin‐53* depletion and NuRD mutants does not further increase the muscle defect (Figure S2C), these observations suggest that *lin‐53* muscle phenotype is caused by impairment of the NuRD complex. To test whether LIN‐53 physically associates with the NuRD complex in muscles, we performed co‐immunoprecipitation experiments coupled to mass spectrometry (IP‐MS) using muscle‐specific expression of FLAG‐tagged LIN‐53. Our muscle‐specific IP‐MS results revealed that LIN‐53 interacts solely with chromatin regulators that are part of the NuRD complex (Figure S2D, Table S2), suggesting that LIN‐53 associates with NuRD in muscles.

Next, we tested whether loss of the NuRD complex would also phenocopy the short lifespan of *lin‐53* mutants. Surprisingly, depletion of NuRD members does not affect the lifespan of *C. elegans* but tends to rather increase lifespan as seen for *let‐418* mutants (Figure 2c) or result in early lethality as seen for *dcp‐66 (gk370)* (Figure S2D) due to pleiotropic effects on organ function as described previously (Maeda, Kohara, Yamamoto, & Sugimoto, 2001). This observation is in agreement with a previous report, showing that *let‐418* mutants display an extended lifespan (De Vaux et al., 2013). Hence, in muscles, LIN‐53 operates as part of the NuRD complex to establish and maintain muscle integrity but does not seem to function through the NuRD complex to ensure a normal lifespan.

### 2.4 Short lifespan of *lin‐53* mutants is phenocopied by *sin‐3* mutants

**Figure 3:**
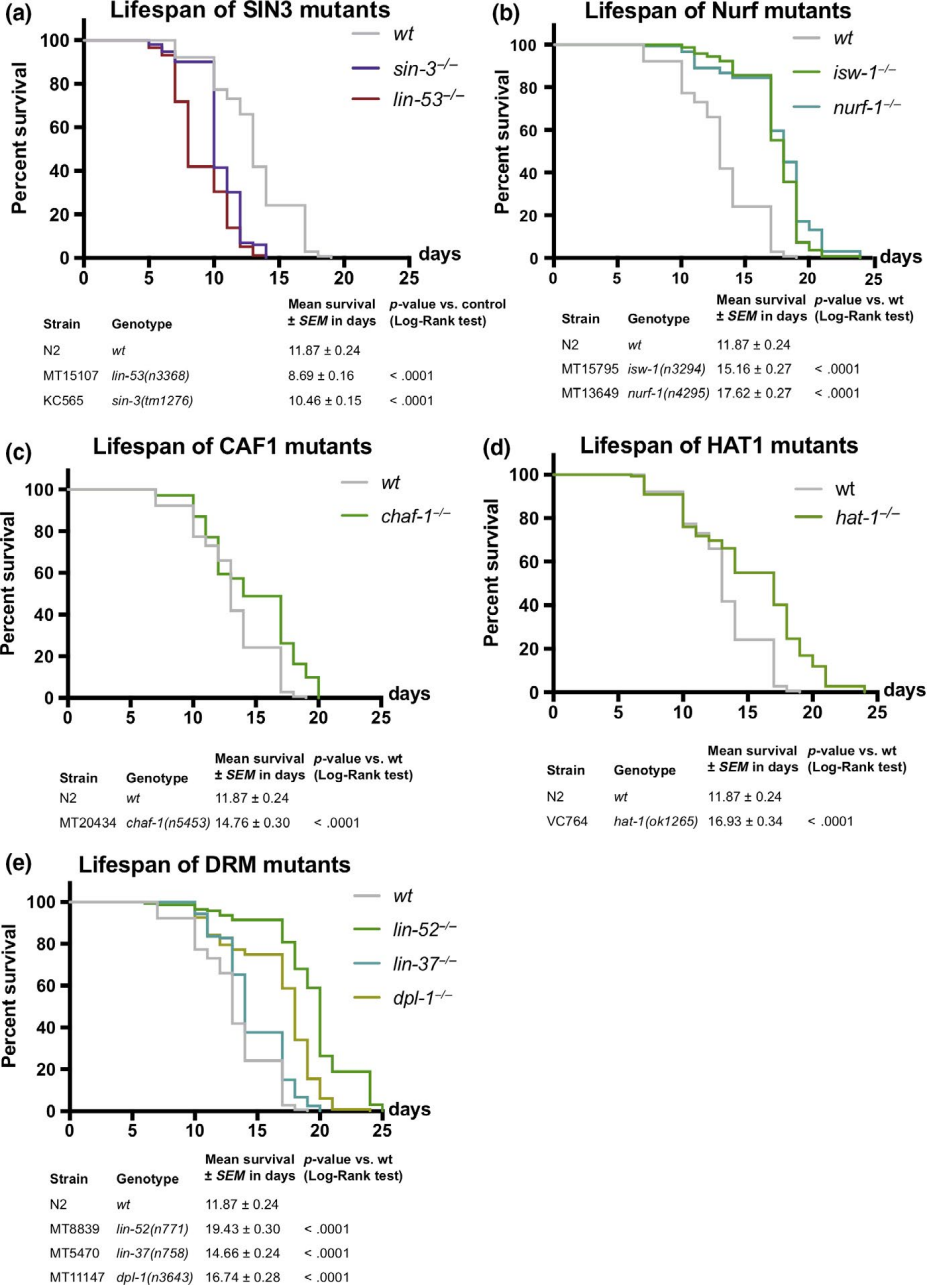
Lifespan of mutants for LIN‐53 interactors. (a) Depletion of Sin3 complex member SIN‐3 leads to shortened lifespan (violet line; mean lifespan 10.46 ± 0.15 days, *p*‐value < .0001; compared with control; gray line; mean lifespan 13.66 ± 0.26 days). Survival analysis was carried out using Kaplan–Meier estimator, and *p*‐value was calculated using log‐rank test. The experiment was done at least three times with at least 40 animals per repeat. (b–e) Lifespan analysis of different mutants for the DRM, Nurf, CAF1, and HAT1 complex. Survival analysis was carried out using Kaplan–Meier estimator, and *p*‐value was calculated using log‐rank test. The experiment was done at least three times with at least 40 animals per repeat

The observation that LIN‐53 as part of the NuRD complex is required for muscle maintenance but not lifespan regulation suggested that LIN‐53 links healthspan with lifespan maintenance through different chromatin‐regulating complexes. To identify through which complex LIN‐53 maintains normal lifespan, we screened for a phenocopy of the short lifespan as seen in *lin‐53* mutants, using available mutants of known LIN‐53‐interacting factors (Figure 3). A partial phenocopy of the short lifespan as seen for *lin‐53* mutants was only detectable in *sin‐3* mutants (Figure 3a), but not upon loss of other known genes encoding a LIN‐53‐interacting protein (Figure 3b‐e). In *C. elegans*, the *sin‐3* gene encodes the core subunit of the Sin3 chromatin‐regulating complex, indicating that, in *lin‐53* mutants, the integrity of the Sin3 complex might be affected, thereby causing the observed shortening of lifespan. This conclusion is further supported by the fact that lifespan is not further decreased upon depletion of *lin‐53* in *sin‐3* mutants arguing that both factors are involved in the same regulatory context (Figures S3A–D). Hence, LIN‐53 and SIN‐3 cooperate to ensure normal lifespan in *C. elegans*.

In summary, LIN‐53 is interacting with the NuRD complex in muscles where its loss leads to a disruption of muscle integrity accompanied by locomotion defects. While affecting the NuRD complex does not lead to a short lifespan, loss of the Sin3 core subunit shortens lifespan, suggesting that LIN‐53 regulates muscle homeostasis as part of the NuRD complex independently of lifespan regulation, which occurs through the Sin3 complex.

### 2.5 Transcriptome of *lin‐53* mutants shows mis‐regulated metabolic genes

**Figure 4:**
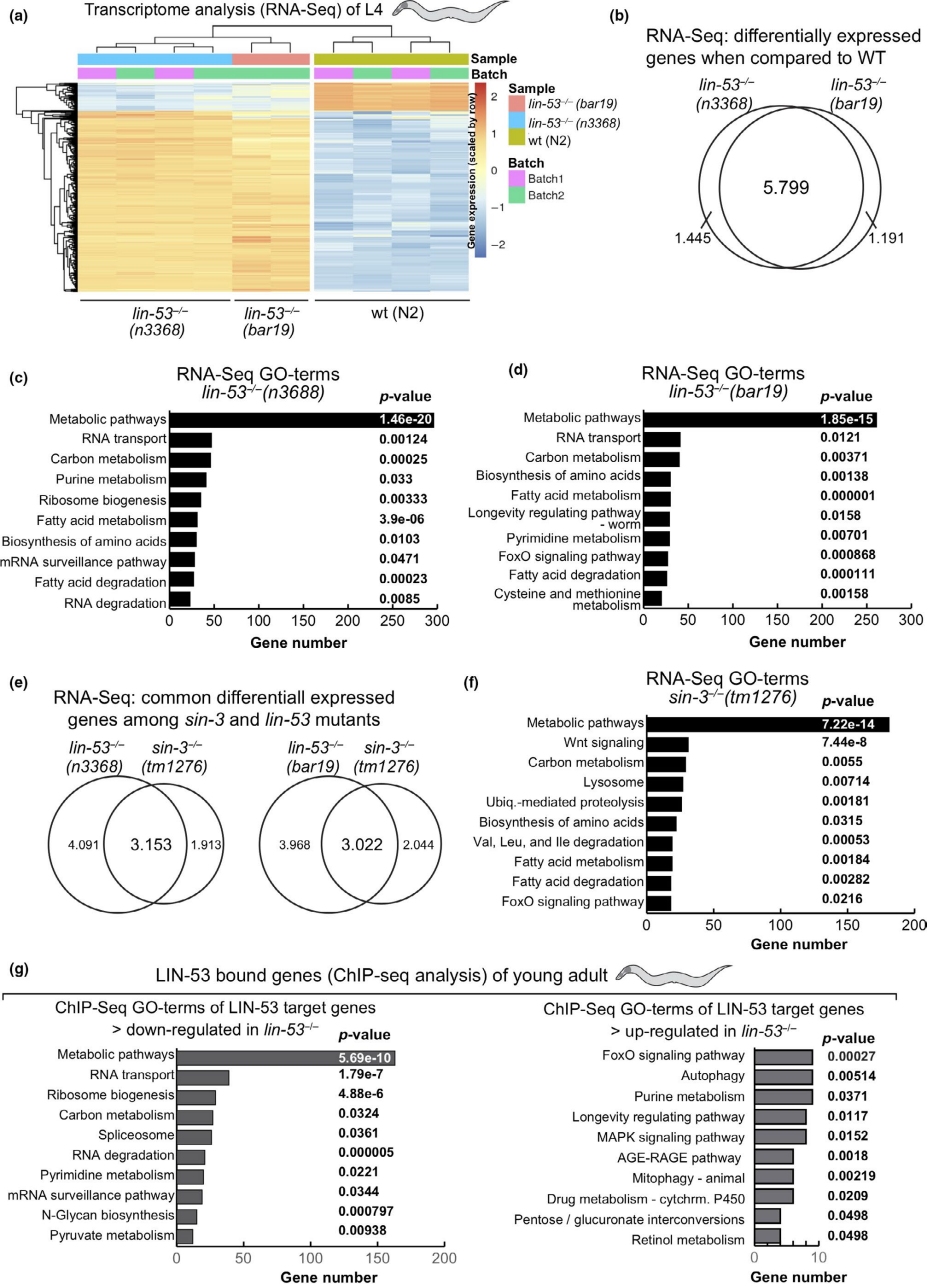
Differentially expressed genes in *lin‐53* and *sin‐3* mutants. (a) Heat map of the normalized expression values (VST) of the top 100 genes with highest variance across samples in *lin‐53(n3368)* and *lin‐53(bar19)* mutants compared with control animals. (b) Venn diagram of differentially expressed genes in *lin‐53(n3368)* and *lin‐53(bar19)* mutants showing more than 5,000 overlapping genes. (c–d) Gene Ontology (GO) term analysis based on KEGG pathways of *lin‐53* mutants compared with control animals using PANTHER. Mainly genes involved in metabolic processes are affected upon loss of *lin‐53*. (e) Venn diagram of differentially expressed genes in *lin‐53* and *sin‐3* mutants. (g) GO term analysis of ChIP‐seq results using PANTHER GO‐Slim biological processes (KEGG pathways) for genes that are bound by LIN‐53 and are either upregulated or downregulated

The shortened lifespan upon loss of LIN‐53 suggested that specific molecular pathways might be affected in *lin‐53* mutants. In order to examine this possibility, we performed whole‐transcriptome sequencing (RNA‐seq) and used both *lin‐53* mutant backgrounds *n3668* (balanced) and *bar19* (CRISPR allele) (Figure 4a). Our analysis revealed that 5.799 genes are differentially expressed in both *lin‐53* mutant backgrounds when compared to the transcriptome of wild‐type N2 animals (Figures 4b and S4A). A number of muscle‐related genes such as *hlh‐1*, *unc‐120*, *unc‐52*, and *myo‐3* are mis‐regulated, which corresponds to the described motility defects in *lin‐53* mutants (Figure S4C). Interestingly, in both *lin‐53* mutant backgrounds GO analysis (KEGG pathways) revealed a strong enrichment for differentially expressed genes that play a role in metabolic pathways (Figures 4c, d, and S4D, E). Since loss of SIN‐3 phenocopies the short lifespan of *lin‐53* mutants, we also performed RNA‐seq analysis of the *sin‐3(tm1276)* mutant background (Figure 4e). Compared to *lin‐53* mutants, more than 50% of the differentially expressed genes in *sin‐3* mutants overlap with those detected in both *lin‐53* mutant backgrounds (Figure 4e). Strikingly, GO analysis revealed a strong enrichment for genes that play a role in metabolic pathways also for *sin‐3* mutants (Figure 4f), suggesting that LIN‐53 cooperates with SIN‐3 in order to regulate metabolism. To elucidate whether LIN‐53 might directly be involved in regulating the expression of “metabolic” genes, we performed chromatin immunoprecipitation with subsequent sequencing (ChIP‐seq) using anti‐LIN‐53 antibody (Figures 4g and S4B, C). The ChIP‐seq analysis revealed that the primary enriched pathway for genes, which are bound by LIN‐53 and become downregulated upon loss of LIN‐53, is implicated in metabolic pathways (Figures 4g, S4D, E). This finding further corroborates the notion that LIN‐53 is important for maintaining expression of genes that are important for metabolic processes. Since it is well established that metabolome alterations have a significant impact on aging (reviewed in Peleg, Feller, Ladurner, and Imhof (2016) and Finkel (2015)), we propose that LIN‐53 is required for normal lifespan of *C. elegans* because it is maintaining the expression of genes that ensure a wild‐type metabolome.

### 2.6 Loss of *lin‐53* leads to decreased levels of trehalose

**Figure 5:**
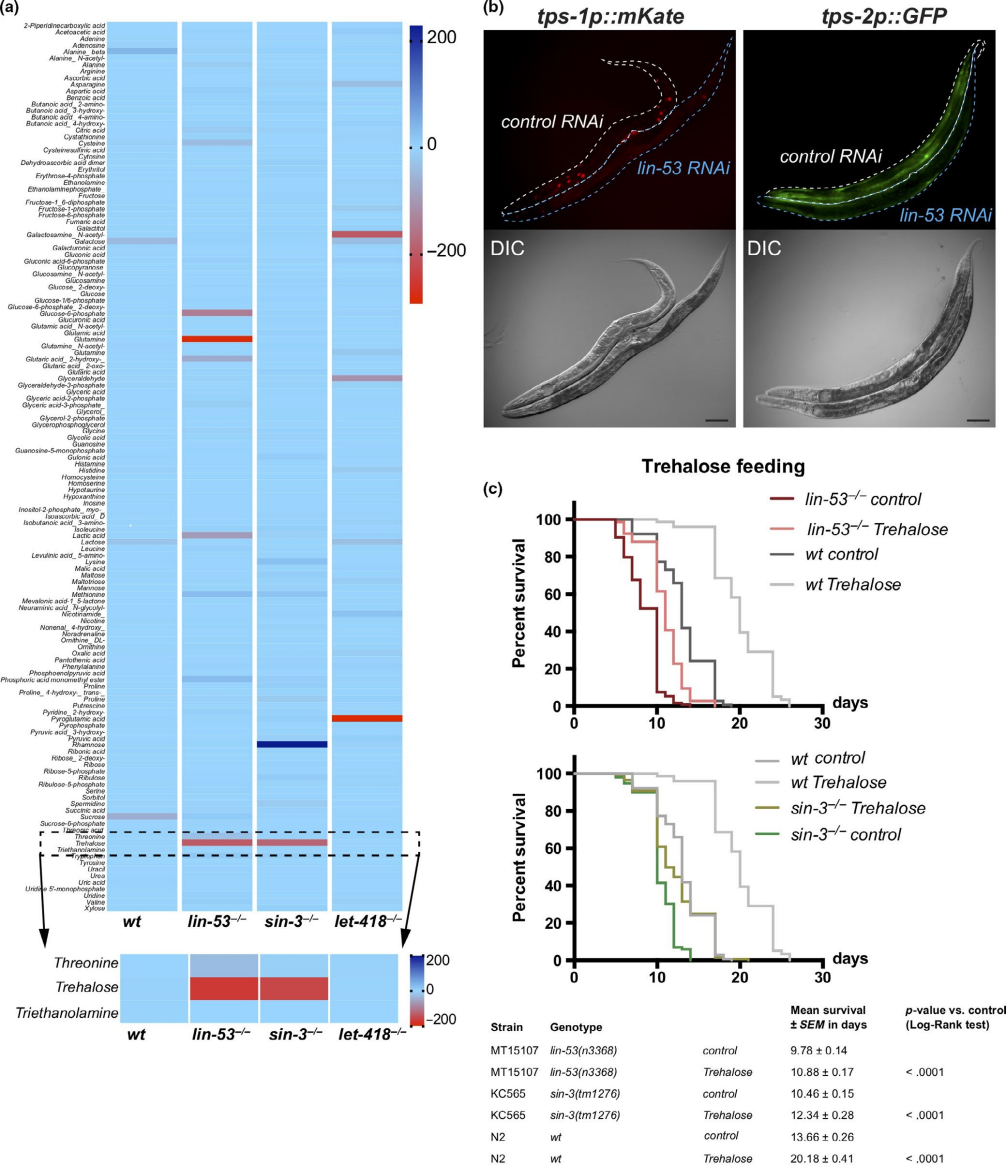
Metabolomic analysis reveals decreased trehalose biosynthesis in mutants. (a) Metabolomic analysis of wt, *lin‐5^−/−^*, *sin‐3^−/−^,* and *let‐418^−/−^* mutants. 215 different metabolites were detected using GC‐MS. The data were z‐transformed and plotted as a heat map. Trehalose is decreased in *lin‐53* and *sin‐3* mutants, but not changed in wt and long‐lived *let‐418* mutants. 2 biological repeats were analyzed. (b) Depletion of *lin‐53* leads to decreased expression of *tps‐1p::mKate* and *tps‐2p:.GFP.* Control and *lin‐53* RNAi‐treated worms were mounted next to each other allowing direct comparison. Upon *lin‐53* RNAi, animals show a decrease in expression of both reporters. (c) Short lifespan of *lin‐53* and *sin‐3* mutants is partially rescued after feeding with trehalose (mean lifespan of *lin‐53* mutants on trehalose 10.98 ± 0.13 days; *lin‐53* mutants 8.58 ± 0.13 days, *p*‐value < .0001; mean lifespan of *sin‐3* mutants on trehalose 12.34 ± 0.28 days; *sin‐3* mutants 10.46 ± 0.15 days, *p*‐value < .0001). The experiments were carried out three times with at least 40 animals scored per repeat. Survival analysis was done using Kaplan–Meier estimator, and *p*‐value was calculated using log‐rank test.

Next, we aimed to assess whether loss of LIN‐53 leads to specific changes in the metabolome as suggested by the transcriptome and ChIP‐seq analyses. We examined the metabolome of *lin‐53* and *sin‐3* mutants at the young adult stage using gas chromatography coupled to mass spectrometry (GC‐MS) and MS data analysis using Maui‐SILVIA (Kuich, Hoffmann, & Kempa, 2014) (also see methods) (Figure 5a, Table S3). Wild‐type animals were used as a control, as well as *let‐418* mutants (NuRD complex). Since *let‐418* mutant animals do not have a shortened lifespan (Figure 2c), metabolites that change in *lin‐53* and *sin‐3* mutant animals*,* but not in *let‐418* mutants, are likely to be implicated in the short lifespan phenotype upon loss of *lin‐53* or *sin‐3*. The unique metabolite, which showed such a pattern, was the glucose disaccharide trehalose. Trehalose levels are decreased in *lin‐53* and *sin‐3* mutants but not in *let‐418* mutants (Figure 5a). Interestingly, it has previously been shown that decreased trehalose levels lead to a shortened lifespan in *C. elegans* (Honda et al., 2010) (Seo et al., 2018), suggesting that reduced trehalose levels in *lin‐53* and *sin‐3* mutants may cause the observed short lifespan of these animals. Impaired maintenance of trehalose levels is also reflected by the fact that reporter expression for the trehalose‐6‐phosphate synthase‐encoding genes *tps‐1* and *tps‐2* (Honda et al., 2010), which are essential for trehalose synthesis, is reduced upon knockdown of *lin‐53* (Figure 5b, and Figure S5A). The downregulation of *tps‐1* in short‐lived *lin‐53* and *sin‐3* mutants, but not in long‐lived *lin‐40* and *let‐418* mutants, was further confirmed by qRT–PCR (Figure S5B).

To provide further evidence that trehalose reduction contributes to shortening the lifespan in *lin‐53* and *sin‐3* mutants, we tested whether feeding of trehalose would alleviate the aging phenotype (Figures 5c and S5C). Replenishing *lin‐53* and *sin‐3* mutants with trehalose by feeding resulted in extended lifespans compared with unfed mutants (Figure 5c), indicating that reduced levels of trehalose play a role in shortening the lifespan upon loss of *lin‐53*. Additionally, the observation that LIN‐53 binds to the genomic sites of *tps‐1* and *tps‐2* upon adulthood indicates that LIN‐53 may regulate both genes directly during aging (Figure S5D).

### 2.7 Loss of LIN‐53 affects the insulin signaling (IIS) pathway

Recently, it has been demonstrated that the insulin/IGF1 signaling (IIS) pathway is important to promote the benefits of trehalose in the context of lifespan maintenance (Seo et al., 2018). It is important to note in this context that animals carrying loss‐of‐function alleles of the *daf‐2* gene, which encodes the IIS receptor, were identified as one of the first mutants with significantly extended lifespans (reviewed in Kenyon (2011)) and it has been reported that trehalose synthesis is upregulated in *daf‐2* mutants (Hibshman et al., 2017; Honda et al., 2010).

**Figure 6:**
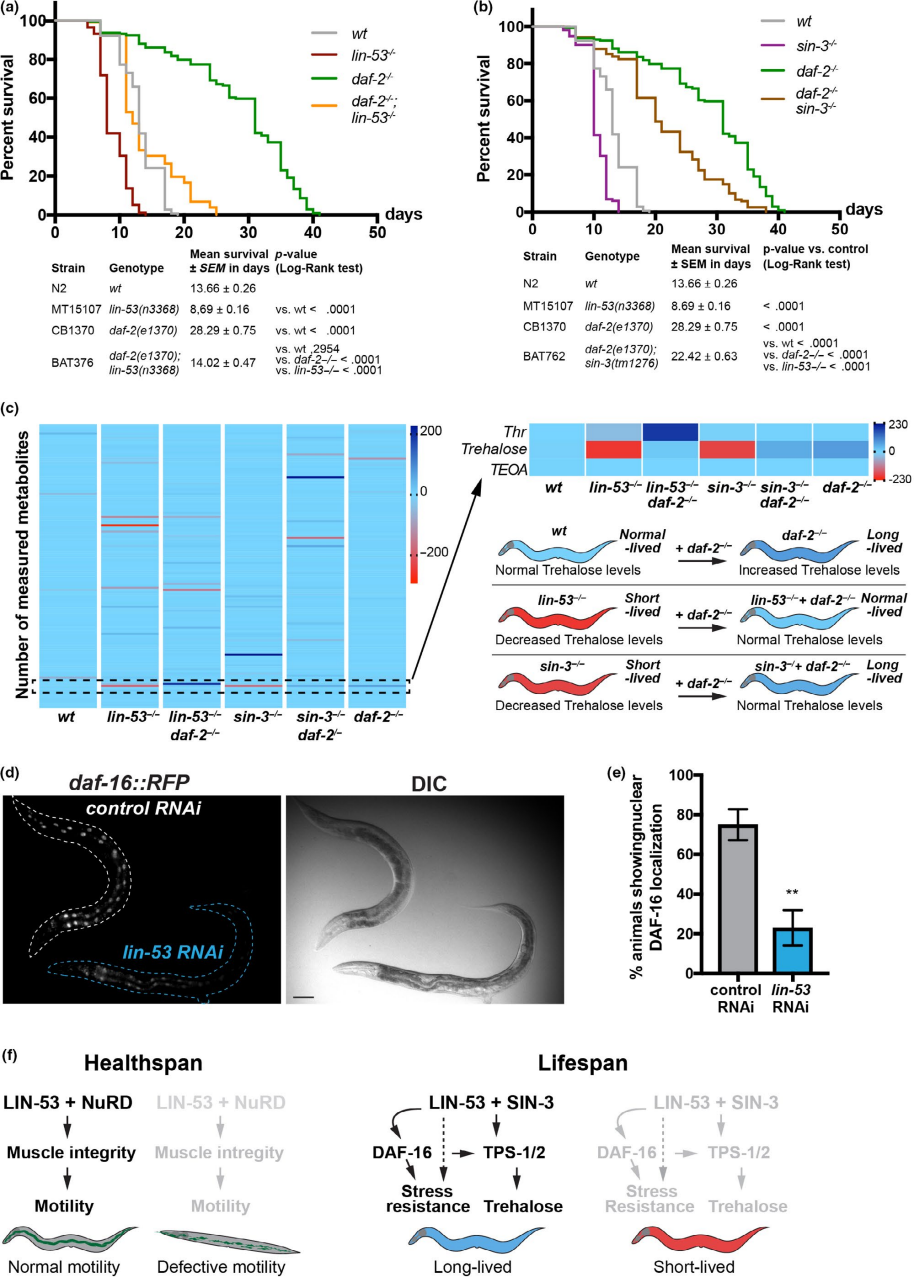
The Insulin/IGF1 receptor mutant *daf‐2(e1370)* restores trehalose levels. (a) The short lifespan of *lin‐53* mutants is partially rescued by *daf‐2*. wt animals (gray line; mean lifespan 13.66 ± 0.26 days), *lin‐53* mutants (red line; mean lifespan 8.69 ± 0.16 days), *daf‐2* mutants (green line; mean lifespan 28.29 ± 0.75 days), and *daf‐2; lin‐53* double mutants (orange line; mean lifespan 14.02 ± 0.47 days). Triplicate experiments were carried out with 40 animals per repeat. Survival analysis was carried using Kaplan–Meier estimator, and *p*‐value was calculated using log‐rank test. (b) As seen for *lin‐53* mutants, short lifespan of *sin‐3* mutants is suppressed by the *daf‐2(e1370)* mutation. wt animals (gray line; mean lifespan 13.66 ± 0.26 days), *sin‐3* mutants (violet line; mean lifespan 10.46 ± 0.15 days), *daf‐2* mutants (green line; mean lifespan 28.29 ± 0.75 days), and *daf‐2 sin‐3* double mutants (brown line; mean lifespan 22.42 ± 0.63 days). Triplicate experiments were carried out with 40 animals per repeat. Survival analysis was carried using Kaplan–Meier estimator, and *p*‐value was calculated using log‐rank test. (c) Metabolomic analysis of wt, *lin‐53^−/−^*, *daf‐2^−/−^; lin‐53^−/−^, daf‐2^−/−^; sin‐3^−/−^*, and *sin‐3^−/−^* mutants. Trehalose levels are increased in long‐lived *daf‐2(e1370)* mutants. The GC‐MS data were z‐transformed and plotted in a heat map. Trehalose is increased in *daf‐2* and restored in *daf‐2; lin‐53* double mutants as well as *sin‐3; daf‐2* double mutants. 2 biological repeats were analyzed. (d) Loss of *lin‐53* leads to decreased nuclear localization of DAF‐16. The translational *daf‐16d::RFP* reporter was grown on *lin‐53* and control RNAi. (e). Quantification of animals showing decreased nuclear *daf‐16d::RFP* upon *lin‐53* RNAi. Statistics are based on unpaired *t*‐test; **p‐value < .05. (F) Model summarizing the findings of LIN‐53 implication in lifespan and healthspan regulation

In order to test whether LIN‐53 is implicated in regulating trehalose levels via the IIS pathway, we first assessed the lifespan of *lin‐53(n3368)*; *daf‐2(e1370)* double mutants (Figure 6a). While the *lin‐53(n3368)*; *daf‐2(e1370)* double mutants live longer than *lin‐53* mutants alone, which is comparable to the lifespan of wild‐type animals, they live significantly shorter than *daf‐2* mutants alone suggesting a requirement for LIN‐53 in IIS pathway‐mediated lifespan extension (Figure 6a). Similar results were obtained when comparing lifespans of the double *sin‐3(tm1276); daf‐2(e1370)* animals with the respective single mutants (Figure 6b). These observations suggested that the previously reported increase of trehalose levels in *daf‐2* mutants (Honda et al., 2010) might compensate for the diminished trehalose levels upon loss of LIN‐53 and SIN‐3. To test this assumption, we analyzed the metabolome of *lin‐53(n3368)*; *daf‐2(e1370)* as well as *sin‐3(tm1276); daf‐2(e1370),* and found that trehalose levels in both double mutants are similar to that of wild‐type animals (Figure 6c). We therefore concluded that *daf‐2* mutants suppress the short lifespan of *lin‐53* and *sin‐3* mutants by counteracting the trehalose deprivation upon loss of LIN‐53 or SIN‐3.

The FOXO transcription factor DAF‐16 is a critical effector of the IIS pathway downstream of DAF‐2 and plays an important role in lifespan regulation (Kwon, Narasimhan, Yen, & Tissenbaum, 2010; Lin, Hsin, Libina, & Kenyon, 2001; Tissenbaum, 2018). Among others, targets of DAF‐16 are the trehalose 6‐phosphate synthases TPS‐1 and TPS‐2 in *C. elegans* (Honda et al., 2010), which we show to also be targeted by LIN‐53 (Figure S5D). Since RNAi against *lin‐53* caused decreased levels of *tps‐1* and *tps‐2* reporter expression, we wondered whether loss of LIN‐53 has an effect on DAF‐16 activity. We assessed the expression of translational reporter *daf‐16d::RFP* (Kwon et al., 2010). *lin‐53* RNAi caused decreased nuclear DAF‐16 levels based on the assessed reporter lines (Figure 6d and e), suggesting that LIN‐53 is required to ensure sufficient DAF‐16d levels in the nucleus.

The effect on DAF‐16 localization prompted us to test whether *lin‐53* mutants suffer from increased stress since DAF‐16 is important for stress tolerance (Tissenbaum, 2018). We used the *gst‐4p::gfp* reporter (Tawe, Eschbach, Walter, & Henkle‐Dührsen, 1998) (Leiers et al., 2003) as an indicator for oxidative stress in *lin‐53* mutants and detected increased expression indicating elevated oxidative stress in animals that lost *lin‐53* (Figure S6A). Furthermore, treatment with the oxidative stress‐inducing drug paraquat resulted in death within 5 hr, suggesting that *lin‐53* mutants cannot tolerate any further oxidative stress (Figure S6B). However, the high sensitivity to increased oxidative stress by paraquat could not be suppressed by the *daf‐2* mutation (Figure S6B), indicating that the oxidative stress in *lin‐53* mutants might be caused in a *daf‐16*‐independent manner. Similar results were obtained upon heat stress treatment of *lin‐53* mutants and *lin‐53(n3368);daf‐2(e1370)* double mutants (Figure S6C).

Overall, our findings suggest that LIN‐53 maintains sufficient trehalose levels to ensure a normal lifespan in conjunction with SIN‐3 and this maintenance has interplay with the IIS pathway in *C. elegans*. Additionally, LIN‐53 is required to ensure tolerance such as against oxidative or heat stress, but the short lifespan in *lin‐53* mutants can be unlinked from the observed muscle defects: LIN‐53 is interacting with the NuRD complex to maintain muscles and proper motility but ensures normal lifespan via the Sin3 complex (Figure 6f). Loss of LIN‐53 or SIN‐3 leads to diminished levels of the disaccharide trehalose, which is required for a normal lifespan (Honda et al., 2010; Seo et al., 2018). These findings suggest that the histone chaperone LIN‐53 is a critical chromatin regulator linking the epigenetic regulation of lifespan with muscle development and integrity, which is an important aspect of healthspan maintenance.

## 3 DISCUSSION

Recent studies revealed epigenetic factors as an emerging group of aging regulators that control gene expression at the level of chromatin (reviewed in Brunet and Rando (2017)). For instance, the ASH‐2 trithorax complex regulates aging in *C. elegans* by catalyzing histone H3 methylation at lysine residue 4 (K4) (Greer et al., 2010) and loss of epigenetic regulation in mouse hematopoietic stem cells accelerates aging (Chambers et al., 2007). Other examples include the sirtuin family of conserved NAD(+)‐dependent enzymes that can deacetylase different protein targets including histones and TFs such as DAF‐16 in *C. elegans*, which are also modulated by acetyltransferases such as CBP‐1 (CBP/p300) reviewed by Chang and Guarente (2014), Houtkooper et al. (2012), Tissenbaum (2018) and Denzel et al. (2019).

In the context of aging regulation, one important aspect is whether healthspan and lifespan are intimately linked. Meaning, should we expect that animals or humans with longer lifespans would also be healthy for a longer time? Interestingly, a recent study by the research group of Heidi Tissenbaum suggests that lifespan and healthspan can be unlinked in *C. elegans* (Bansal, Zhu, Yen, & Tissenbaum, 2015). For instance, the authors showed that in many cases, when lifespan is extended, there is an increase in the time for which animals live in a frail state (Bansal et al., 2015). As many aging‐regulating pathways are evolutionarily conserved, an unlinking of lifespan and healthspan is conceivable also in higher organisms. Our findings described in this study corroborate this notion as the histone chaperone LIN‐53 is highly conserved in metazoan species (known as RBBP4/7 and CAF‐1p48 in mammals; Harrison et al., 2006). We found that overexpression of the chromatin regulator LIN‐53 specifically in *C. elegans* muscles rescued the *lin‐53* null‐mutant phenotype with regard to the muscle and motility defects. Strikingly, high levels of LIN‐53 in muscles of wild‐type animals are beneficial for muscle function as we demonstrate using two independent lines overexpressing LIN‐53 with or without a GFP fusion. Motility remained in a healthy juvenile state in aged animals, indicating that LIN‐53 is required for motility during late stages of development and promotes health muscles in aging adult animals. However, while muscle‐specific LIN‐53 overexpression reconstituted muscle integrity in aging *lin‐53* mutants, the lifespan of these animals remained short, which suggests that the effects of LIN‐53 on muscle development and maintenance versus its role in lifespan regulation are separable. Our finding that LIN‐53 associates with the NuRD complex in order to establish muscle integrity during late postembryonic development and maintain muscle health throughout adulthood, while its role in lifespan homeostasis is mediated via the Sin3 complex, confirmed this initial assumption. Notably, mutants for the NuRD subunit LET‐418 have an increased lifespan, but still show a compromised movement, suggesting that loss of the NuRD complex only affects muscle integrity. While the exact molecular mechanism by which LIN‐53 regulates muscle homeostasis remains to be determined, our findings provide an important initial framework for elucidating LIN‐53’s roles in muscles via the NuRD complex.

With respect to the aging phenotype of *lin‐53* mutants, we found that the shortened lifespan may be caused by loss of the Sin3 complex. Animals deleted for the *sin‐3* gene phenocopy at least partially the short lifespan of *lin‐53* mutants and loss of *lin‐53* appears to have more pleiotropic effects on gene expression than seen for *sin‐3* mutants, which could explain the stronger impact on lifespan in *lin‐53* mutants. Nevertheless, loss of either *lin‐53* or *sin‐3* primarily affects the expression of genes related to metabolic processes prompting us to assess changes in the metabolome in these mutants. Strikingly, our analysis showed that trehalose levels are diminished in both *lin‐53* and *sin‐3* mutants, thereby revealing a possible common factor with regard to impacting lifespan regulation. It is known that decreased trehalose levels result in a shorter lifespan in *C. elegans*, as described earlier (Honda et al., 2010). Our finding that trehalose levels are reconstituted when we combine either *lin‐53* or *sin‐3* mutants with the *daf‐2* mutant background is, therefore, in agreement with previous studies showing that the insulin/IGF1 signaling (IIS) pathway controls trehalose levels and that loss of DAF‐2 results in increased trehalose levels (Honda et al., 2010). Recently, it has been demonstrated that lifespan extension upon increased trehalose levels depends on the FOXO transcription factor DAF‐16, which acts downstream of DAF‐2 (Kenyon, 2011; Seo et al., 2018). DAF‐2 activation triggers phosphorylation of DAF‐16 by the PI3‐kinase AGE‐1 (Morris, Tissenbaum, & Ruvkun, 1996; Wolkow, Muñoz, Riddle, & Ruvkun, 2002) and the serine/threonine kinase PDK‐1 (Landis & Murphy, 2010; Paradis & Ruvkun, 1998), resulting in the inhibition of DAF‐16 translocation to the nucleus (Murphy et al., 2003). Previously identified targets of DAF‐16 include the trehalose synthesis‐regulating genes *tps‐1* and *tps‐2* (McElwee, Bubb, & Thomas, 2003; Pellerone et al., 2003)*.* Hence, it is conceivable that the observed reduction in DAF‐16 levels in the nucleus upon loss of LIN‐53 results in decreased *tps‐1* and *tps‐2* expression, which consequently leads to diminished trehalose levels, thereby causing a short lifespan phenotype. It remains to be determined why DAF‐16 levels decrease in the nucleus upon LIN‐53 depletion. One possible explanation is that LIN‐53 plays a role during nuclear import as it has been shown for its human counterpart RBBP4 in senescent cells (Tsujii et al., 2015). It is possible that loss of LIN‐53 alters the nuclear import machinery leading to the retention of essential DAF‐16 proteins in the cytoplasm.

The observation of elevated oxidative stress in animals that lost *lin‐53* seems to be in a *daf‐16*‐independent manner as the high sensitivity to increased oxidative stress by paraquat could not be suppressed by the *daf‐2* mutation. We cannot explain how exactly oxidative stress is caused upon loss of LIN‐53, but the impairment of multiple genes belonging to metabolic pathways may provide a clue for further studies.

Interestingly, our RNA‐seq did not indicate a dramatic difference for *tsp‐1/2* expression. Nevertheless, the strength of our study lies in the multilayered analysis ranging from transcriptomics and genomics down to metabolomics in order to identify the physiologically relevant changes upon loss of *lin‐53*. The metabolomic analysis provided us with a stringent and filtered metabolic process (trehalose synthesis), which is relevant for the observed lifespan phenotype upon loss of *lin‐53*. Strikingly, the changes in *tps‐1/2* expression upon *lin‐53* depletion were evident when examining the *tps‐1* and *tps‐2* reporter lines. The fact that LIN‐53 binds to the genomic sides of both genes upon adulthood indicates a rather late onset of direct regulation by LIN‐53.

Additionally, we cannot exclude the possibility that trehalose levels might increase via the glyoxylate shunt (GS) as we can see that the gene *icl‐1*, which encodes for an enzyme with isocitrate lyase and malate synthase activity required for the GS (Erkut, Gade, Laxman, & Kurzchalia, 2016), shows increased transcript levels in *lin‐53* mutants.

A conserved role for LIN‐53 in aging regulation is conceivable because its human homologs RBBP4 and RBBP7 have been implicated in Hutchinson–Gilford progeria syndrome (HGPS), which leads to premature aging (Pegoraro et al., 2009). HGPS belongs to laminopathic disorders caused by mutations in genes encoding for lamin A/C or for other nuclear lamina proteins such as emerin (Zaremba‐Czogalla, Dubińska‐Magiera, & Rzepecki, 2010). In primary dermal fibroblasts of HGPS patients, RBBP4/7 levels are significantly reduced, which is also the case in fibroblasts from aged human beings (Pegoraro et al., 2009). However, in their study Pegoraro et al. proposed that the premature aging disorder is caused by the loss of functional NuRD complexes due to reduced levels of RBBP4/7 (Pegoraro et al., 2009). While we do not see premature aging upon depletion of specific NuRD subunits such as LIN‐40, LIN‐61, or LET‐418 in *C. elegans,* we identified the Sin3 complex to be relevant for the aging phenotype upon loss of LIN‐53. We speculate that the Sin3 complex might also play a role during aging regulation in other species as it has also been shown by a previous study in *Drosophila* that knockdown of the Sin3A gene causes a shortened lifespan (Barnes et al., 2014).

In humans, LIN‐53 homologs might link aging regulation with healthspan as we see it in the *C. elegans*. Laminopathies such as the Emery–Dreifuss muscular dystrophy (EDMD) diminish muscle maintenance and motility in patients (Zaremba‐Czogalla et al., 2010). Laminopathies in general might affect RBBP4/7 levels as shown in the laminopathic disorder HGPS, thereby recapitulating the phenotypes of *lin‐53* mutant worms. Hence, reduced levels of LIN‐53 and its homolog RBBP4/7 might lead to premature aging and impaired maintenance of muscle integrity in *C. elegans* as well as humans. Interestingly, it has recently been revealed that the decline of RBBP4 in humans causes cognitive aging and memory loss (Pavlopoulos et al., 2013). Hence, it is conceivable that the conserved histone chaperone LIN‐53 links lifespan regulation with aspects of healthspan maintenance also in higher organisms, which can have important implications for human health during aging.

## 4 EXPERIMENTAL PROCEDURES

List of strains used in this study.

**Table:** 

Strain names	Genotypes	References
N2	*Wt*	CGC
MT15107	*lin‐53(n3368)I/hT2 [bli‐4(e937) let‐?(q782) qIs48] (I;III)*	CGC
CB1370	*daf‐2(e1370)*	CGC
BAT376	*daf‐2(e1370); lin‐53(n3368)*	This study
BAT1883	*lin‐53(n3368); barEx974 [baf‐1p::GFP::lin‐53::2xFLAG]*	This study
BAT703	*lin‐53(n3368); barIS87 [myo‐3p::lin‐53::2xFLAG]*	This study
BAT729	*lin‐53(n3368); barIS87 [myo‐3p::lin‐53::2xFLAG]*	This study
HX103	*chd‐3(eh4)*	CGC
VC924	*dcp‐66(gk370)*	CGC
MT14390	*let‐418(n3536)*	CGC
VC660	*lin‐40(ok905)*	CGC
MT12833	*lin‐61(n3809)*	CGC
MT15795	*isw‐1(n3294)*	CGC
MT13649	*nurf‐1(n4295)*	CGC
MT8839	*lin‐52(n771)*	CGC
MT5470	*lin‐37(n758)*	CGC
MT11147	*dpl‐1(n3643)*	CGC
MT20434	*chaf‐1(n5453)*	CGC
KC565	*sin‐3(tm1276)*	CGC
VC764	*hat‐1(ok1265)*	CGC
BAT762	*daf‐2(e1370); sin‐3(tm1276)*	This study
BAT1368	*myo‐3p::lin‐53_IR::2xNLS::tagRFP*	This study
BAT1265	*myo‐3p::lin‐53::GFP::2xFLAG::SL2::tagRFP line 2*	This study
BAT1982	*tps‐1p::mKATE::H2B*	This study
BAT1943	*barSi23 [baf‐1p::GFP::lin‐53::2xFLAG::SL2::NLS::tagRFP]*	This study
BC18476	*tps‐2p::GFP*	CGC
WM118	*rde‐1(ne300) V; neIs9 X.*	CGC
VP303	*rde‐1(ne219) V; kbIs7[nhx‐2prom::rde‐1 + rol‐6(su1006)]*	CGC
NR222	*rde‐1(ne219) V; kzIs9[pKK1260(lin‐26p::nls::GFP) + pKK1253(lin‐26p::rde‐1) + pRF6(rol‐6(su1006)]*	CGC

List of RNAi clones used in this study.

**Table:** 

Gene names	Derived from
Empty vector	Addgene
*lin‐53*	Tursun et al. (2011)
*chd‐3*	Chromatin 2.0 library from (Hajduskova et al., 2019)
*dcp‐66*	Chromatin 2.0 library from (Hajduskova et al., 2019)
*hda‐1*	Chromatin 2.0 library from (Hajduskova et al., 2019)
*let‐418*	Chromatin 2.0 library from (Hajduskova et al., 2019)
*lin‐40*	Chromatin 2.0 library from (Hajduskova et al., 2019)
*lin‐61*	Chromatin 2.0 library from (Hajduskova et al., 2019)

List of primers for qPCR.

**Table:** 

Target	Sequence 5'‐3'
*cdc‐42 fwd*	ATGCAGACGATCAAGTGCGTCGTCG
*cdc‐42 rev qRT*	GTGGATACGATAGAGGCC
*lin‐53 fwd qRT*	GTGTGGGACCTATCTAAGA
*lin‐53 rev*	TTACTGTTGTCTCTCTACCAC
*tps‐1 fwd*	AGATACGAATTTGCAAGAAAAAGT
*tps‐1 rev*	TCCAGTTTTCGGTTTCTCTCA

### 4.1 Lifespan and locomotion assay

For age synchronization, eggs were put on a plate, which was scored as day 0. If not stated differently, worms were grown until L4 stage at 15°C and then transferred to plates containing 5‐fluoro‐2′‐deoxyuridine (FUDR; 10 worms per plate) and further grown at 25°C. FUDR treatment did not show any effect on the lifespan of wt and *lin‐53* mutants (Figure S1F). The animals were scored daily for survival, and the locomotion was classified (categories A–C) (Herndon et al., 2002). Animals that did not show pharyngeal pumping or respond to podding were scored as dead. For data analysis of the survival, OASIS was used (Han et al., 2016).

### 4.2 Thrashing assay

Age‐synchronized worms were put in a drop of 10 µl M9 buffer, and a video was made using the DinoXcope software in combination with a Dino‐Lite camera. The video was taken with a resolution of 640 × 480 with a frame rate of 24.00 fps for 30 s at normal quality. The calculation of the body bends was carried out using the ImageJ Plugin wrMTrck (Jesper S. Pedersen) with the according settings.

### 4.3 Immunostaining and antibodies

Staining was carried out as previously described (Seelk et al., 2016). In brief, worms were freeze‐cracked after resuspension in 0.025% glutaraldehyde between two frost‐resistant glass slides on dry ice. The animals were fixed using acetone/methanol for 5 min each and washed off into PBS. Afterward, the sample was blocked in 0.25% Triton X‐100 + 0.2% gelatin in PBS and stained. Primary antibodies were diluted in PBS with 0.25% Triton X‐100 + 0.1% gelatin, and the fixed worms were incubated overnight at 4°C. After PBS washes, secondary antibody was added for 3 hr. Worms were mounted with DAPI‐containing mounting medium (Dianova, #CR‐38448) on glass slides after further washing steps. The primary antibodies used were anti‐MHC (1:300; DHSB #5‐6‐s) and anti‐LIN‐53 (1:800, Pineda). As secondary antibodies, Alexa Fluor dyes were applied at 1:1,000 dilution.

For phalloidin staining, the worms were harvested with PBS and fixed with 4% formaldehyde in PBS. For freeze‐cracking, worms were frozen in liquid nitrogen followed by thawing at 4°C for three times. The samples were incubated for 30 min followed by three times washing with PBST for 10 min. Phalloidin rhodamine in PBST was added and incubated for 30 min on room temperature. After a last washing for three times 10 min in PBST, the worms were mounted on slide using mounted with DAPI‐containing mounting medium (Dianova, #CR‐38448). Microscopy was done using the Zeiss Axio Imager 2 fluorescent microscope.

### 4.4 RNA interference

RNA inference was usually carried out as P0, meaning that eggs were put on RNAi plates and the same generation was scored. Worms were bleached and eggs were put on RNAi plates seeded with bacteria expressing dsRNA or carrying an empty RNAi vector and grown at 15°C until they reached L4 stage. If animals were used for a lifespan experiment, 10 animals were put on one RNAi plate containing FUDR and cultured further at 25°C. For monitoring of the muscle structure, worms were grown at 15°C until they reached the stage of interest and analyzed by fluorescent microscopy. For compiling the *lin‐53* interaction partner sublibrary, candidate genes interacting with LIN‐53 were chosen based on a literature search (www.pubmed.com). The library was generated by compiling the clones from the chromatin RNAi sublibrary generated in Hajduskova et al. (2019).

### 4.5 Generation of a *lin‐53* hairpin construct

For generation of a *lin‐53* short hairpin construct (shRNA), the desired fragment was amplified using specific primers to introduce two different restriction sites at both ends of the cDNA of *lin‐53* (Tavernarakis, Wang, Dorovkov, Ryazanov, & Driscoll, 2000). The restriction site is used as an inversion point to ligate two pieces of *lin‐53* together to form an inverted repeat and clone into a plasmid carrying a muscle‐specific promoter to enable expression of the *lin‐53* shRNA in muscles.

### 4.6 Co‐immunoprecipitation with subsequent mass spectrometry (IP‐MS)

Worms were synchronized by bleaching and grown on 5–10 15‐cm plates until L4 stage. Worms were washed off the plates using M9 buffer and freeze‐cracked by adding the worm suspension dropwise to liquid nitrogen, pulverized using a hammer and a bio‐pulverizer for 20–30 times, and afterward ground to a fine powder using a mortar. The powder was thawed and dissolved in 1.5 vol lysis buffer (50 mM HEPES‐KOH (pH 7.6); 1 mM EDTA; 0.25 M LiCl; 1% sodium deoxycholate; 0.5% NP‐40; 100 mM NaCl; 10% glycerol + 1 tablet of Complete in 10 ml of buffer). In order to shear DNA, the sample is sonicated using Bioruptor® device for six times with 30 s ON and 30 s OFF on high settings. The lysate is cleared by spinning and µMACS‐DKYDDDK (Miltenyi Biotec) is added and incubated for 30 min on ice, before the mixture is applied on a magnetic M column. After washing three times with lysis buffer, the samples are eluted using 8 M guanidine hypochloride preheated to 80°C for mass spectrometry (MS). Sample preparation for MS was done as described previously (Hajduskova et al., 2018). Raw MS data were analyzed using MaxQuant Software (Cox & Mann, 2008).

### 4.7 RNA extraction

Whole‐transcriptome sequencing was carried out as previously described (Kolundzic et al., 2018). In brief, RNA was extracted from control, *lin‐53(n3368)*, *lin‐53(bar19),* and *sin‐3(tm1276)* animals using TRIzol (Life Technologies) and guanidinium thiocyanate‐phenol‐chloroform extraction. Adding chloroform to the TRIzol sample leads to a phase separation with the aqueous phase containing the RNA, an interphase and an organic phase containing DNA and proteins. The RNA was further purified from the aqueous phase using isopropanol. For control, *lin‐53(n3368)* and *sin‐3(tm1276)* four biological replicates were prepared, for *lin‐53(bar19)* RNA was extracted from two biological replicates.

### 4.8 qRT–PCR

To analyze gene expression, RNA was first reverse‐transcribed using GoScript Reverse Transcriptase (Promega) using oligo(dT) and random hexamer primers. The qPCR was carried out using Maxima SYBR Green/ROX qPCR Master Mix (2×) according to the manufacturer's instructions. The measurement was done using the CFX96 Touch Real‐Time PCR Detection System from Bio‐Rad. *cdc‐42* was used as a reference gene, and relative expression was calculated using the Livak method (Schmittgen & Livak, 2008).

### 4.9 Whole‐transcriptome sequencing

Library preparation for RNA sequencing was carried out using TruSeq RNA Library Prep Kit v2 (Illumina) according to the manufacturer's instructions. Libraries were sequenced using paired‐end sequencing length of a 100 nucleotides on a HiSeq 4000 machine (Illumina).

### 4.10 Analysis of RNA‐seq data

The RNA‐seq sequencing datasets were processed using the PiGx‐RNA‐seq (Wurmus et al., 2018) pipeline (version 0.0.4), in which the quality of the raw fastq reads was improved using Trim Galore (https://www.bioinformatics.babraham.ac.uk/projects/trim_galore/), and gene‐level expression was quantified using Salmon (Patro, Duggal, Love, Irizarry, & Kingsford, 2017) based on worm transcript annotations from the Ensembl database (version 89). The raw read counts were further processed using RUV function of the RUVseq R package (Risso, Ngai, Speed, & Dudoit, 2014) to remove unwanted variation from the expression data. Covariates discovered using RUVseq was integrated with DESeq2 (Love, Huber, & Anders, 2014) to test for differential expression using a lfcThreshold of 0.5 and false discovery rate of 0.05. The GO term enrichment is calculated using the gProfileR package. The *p*‐values are corrected for multiple testing. The default multiple testing correction method (“analytical”) was used when running the gProfileR’s main enrichment function “gprofiler.”

### 4.11 ChIP‐seq

In brief, in M9 arrested L1 worms were grown on OP50 plates for 40 hr to L4/YA stage at room temperature. Animals were washed three times with M9 and fixed with 2% formaldehyde for 30 min followed by quenching with 0.125 M glycine for 5 min. The samples were rinsed twice with PBS, and 200–300 µl of pellets was snap‐frozen in liquid nitrogen and kept at −80°C. The pellets were washed once with 0.5 ml PBS + PMSF and resuspended in 1 ml FA Buffer (50 mM HEPES/KOH pH 7.5, 1 mM EDTA, 1% Triton X‐100, 0.1 sodium deoxycholate, 150 mM NaCl) + 0.1% sarkosyl + protease inhibitor (Calbiochem) and then dounce‐homogenized on ice with 30 strokes. The samples were sonicated with Bioruptor with the setting of high power, 4°C, and 15 cycles of 30 s on 30 s off. Soluble chromatin was isolated by centrifugating for 15 min at max speed and 4°C. The cellular debris was resuspended in 0.5 FA Buffer + 0.1% sarkosyl + protease inhibitor and sonicated again 15 cycles with the same setting. Isolated soluble chromatin preparations were combined. The immunoprecipitation of LIN‐53 protein was performed overnight in a total volume of 600 μl with 10 µl of LIN‐53 PA58 (polyclonal peptide AB; rabbit, Pineda), while 5% of samples were taken as input. Immunocomplexes with collected with protein A‐sepharose beads (Sigma). The beads were washed with 1 ml of following buffers: twice with FA Buffer for 5 min, FA + 1 M NaCl for 5 min, FA + 0.5 M NaCl for 10 min, TEL Buffer (0.25 M LiCL, 1% NP‐40, 1% sodium deoxycholate, 1 mM EDTA, 10 mM Tris–HCl pH 8.0) for 10 min, and twice with TE Buffer (pH 8.0). DNA–protein complexes were eluted in 250 µl ChIP elution buffer (1% SDS, 250 mM NaCl, 10 mM Tris pH 8.0, 1 mM EDTA) at 65°C for 30 min. The inputs were treated for approx. 3 hr with 20 μg RNase A (Invitrogen). Samples and inputs were incubated with 10 μg of proteinase K for 1 hr and reverse cross‐linked overnight at 65°C. DNA was purified with Qiagen MinElute PCR Purification Kit. Sequencing library preparation was carried out using NEXTflex qRNA‐seq Kit v2 Set A kit according to manufacturer's instructions. Libraries were sequenced using at HiSeq 4000 (Illumina) with 2 x 75 bp.

### 4.12 ChIP‐seq sequencing data processing

The ChIP‐seq sequencing datasets were processed using the PiGx‐ChIP‐seq (Wurmus et al., 2018) pipeline (version 0.0.16), in which the quality of the raw fastq reads was improved using Trim Galore (https://www.bioinformatics.babraham.ac.uk/projects/trim_galore/), processed reads were aligned to the DNA sequence assembly WBcel235 (Ensembl version 89) using Bowtie2 (Langmead & Salzberg, 2012), the peaks were called using MACS2 (Zhang et al., 2008), and the IDR (irreproducible discovery rate) peaks were called using the IDR software (Li, Qunhua Li, Brown, Huang, & Bickel, 2011).

### 4.13 Metabolomic analysis

Wild‐type, *daf‐2(e1370)*, *lin‐53(n3368)*, *sin‐3(tm1276)*, *daf‐2(e1370); lin‐53(n3368), sin‐3(tm1276);daf‐2(e1370), and let‐418(n3536)* animals were analyzed at L4/young adult stage. For sample collection, worms were synchronized by bleaching and transferred to NGM plates containing OP50 as food source. Worms were grown at 15°C until L3/4 stage and then shifted to 25°C until young adult stage. The *pvul* phenotype of *lin‐53*‐deficient worms was taken as an indicator to select for homozygous mutants derived from the balanced strain MT15107. The limited number of available homozygous *lin‐53^−/−^* animals was sufficient for two replicate experiments. Worms were harvested in M9 medium and adjusted to approx. 40 mg of worms per sample. The sample extraction is performed by using methanol::chloroform::water (5:2:1, MCW; 1 ml per 50 mg sample). To lyse worms and immediately bring the metabolites in solution, 500 µl ice‐cold MCW (with cinnamic acid) is added to the frozen worm sample. The sample was transferred to a new tube containing silica beads and lysed using mechanical force with a tissue lyser at 6,500 m/s, 2 × 20 s ON, 5 s OFF for three times. To further solubilize, the lysate was sonicated for 10 min in an ultrasound bath. As much supernatant as possible was taken off the beads and the leftover MCW (*x*–500 µl) was added, the sample was vortexed and shortly incubated on dry ice. After shaking for 15 min, 1,400 rpm at 4°C, 0.5 vol of water was added for phase separation. The sample was vortexed and shaken for 15 min, 1,400 rpm at 4°C. Vortexing was repeated and the sample was spun at max. speed, 4°C for 10 min to ensure phase separation. The polar phase was taken off and further prepared for measurement. The polar phase was dried overnight in a speed vac followed by derivatization. For this, first 10 µl of 40 mg/ml methoxyamine hydrochloride (MeOx) solution in pyridine is added and incubated for 90 min at 30°C with shaking. Afterward, 30 µl N‐methyl‐N‐(trimethylsilyl) trifluoroacetamide (MSTFA) is added to the sample. Per 1,000 µl of MSTFA 10 µl of a standard retention index mixture of different decanes (C17 mix) is dissolved in MSTFA. Everything is incubated at 30°C for 1 hr with shaking. After spinning down for 10 min at full speed, the samples are put into glass vials for the GC‐MS. Cinnamic acid was used as internal standard to monitor sample processing and measurements. Identification of metabolites was performed using an in‐house database and the Golm Metabolome Database as previously described (Kopka et al., 2005). Additionally, the measurement of 110 metabolites was performed in the same batch.

For quantification, mixtures containing 74 metabolites were measured in 8 dilutions within the same batch as previously described (Pietzke, Zasada, Mudrich, & Kempa, 2014). These measurements were used to monitor the dynamic range of the mass spectrometer for a wide range of metabolite classes. The data analysis was carried out using Maui‐SILVIA (Kuich et al., 2014).

## CONFLICT OF INTEREST

None declared.

## AUTHOR CONTRIBUTION

SM and BT designed the study and interpreted the results. SM, AK, BV, SB, IM, and MH conducted experiments and analyzed data. SM and BT wrote the manuscript. BU and AA performed bioinformatic analyses. SK supported metabolomic analysis. BT acquired funding for the project from the ERC. All authors assisted in editing the manuscript.

## Data Availability

To see a detailed description of the downstream analysis of RNA‐seq and ChIP‐seq data, see the GitHub repository: https://github.com/BIMSBbioinfo/Muthel_et_al_lin53_2019.
