# Supplementary material for: The conserved histone chaperone LIN‐53 is required for normal lifespan and maintenance of muscle integrity in Caenorhabditis elegans
Source: Aging Cell. 2019 Aug 9;18(6):e13012. doi: 10.1111/acel.13012 (PMC6826145; doi:10.1111/acel.13012)
Supplement: Supplementary file 1 [file ACEL-18-e13012-s001.pdf]

Figure S1

Müthel et al.

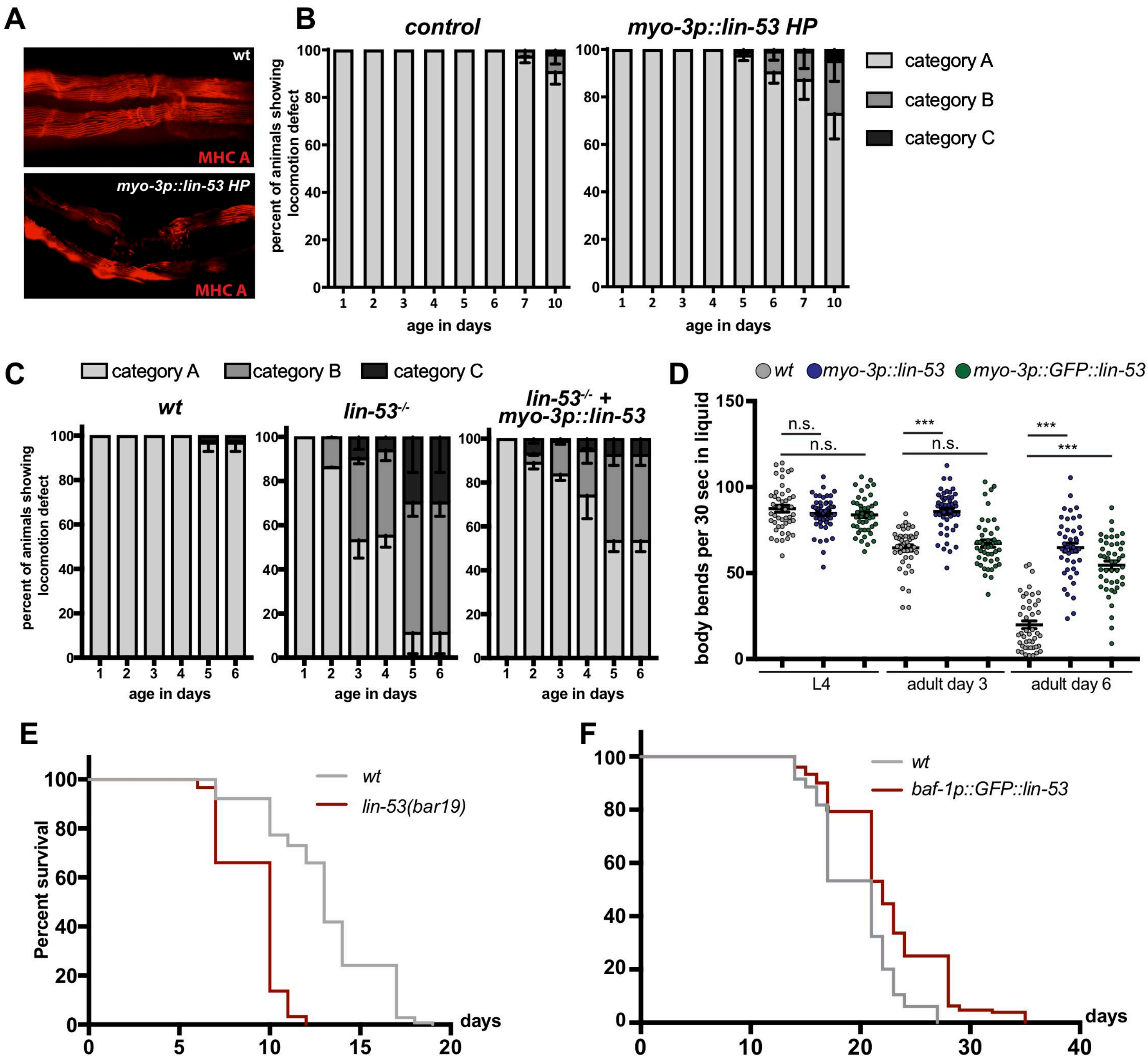

E

Strain	Genotype	Mean survival ± SEM in days	p-value vs. wt (Log-Rank test)
N2	*wt*	13,66 ± 0,26	
MT15107	*lin-53(n3368)*	8,69 ± 0,16	< 0,0001
BAT320	*lin-53(bar19)*	9,12 ± 0,14	< 0,0001

F

Strain	Genotype	Mean survival ± SEM in days	p-value vs. wt (Log-Rank test)
N2	*wt*	19,61 ± 0,32	
BAT1943	*baf-1p::GFP::lin-53*	22,42 ± 0,38	< 0,0001

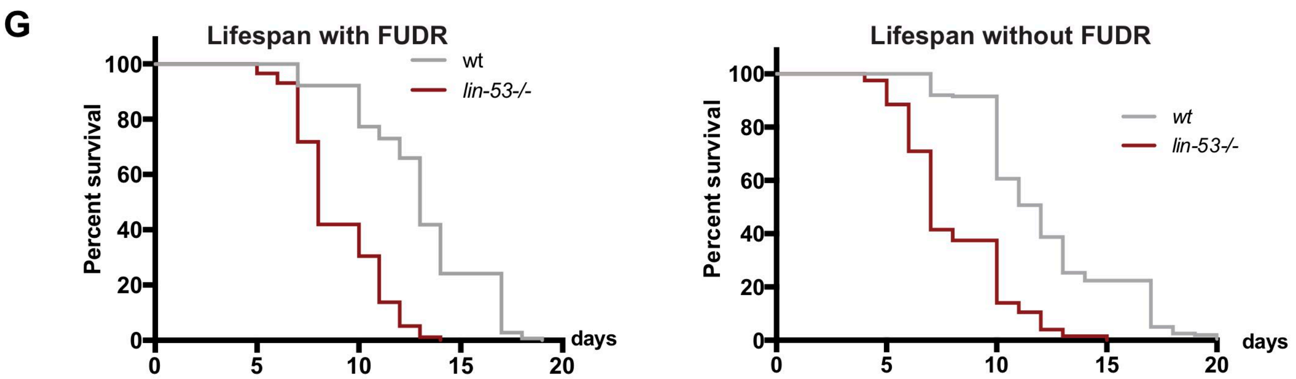

Lifespan with FUDR

Strain	Genotype	Mean survival ± SEM in days	p-value (Log-Rank test)
N2	*wt*	13,66 ± 0,26	
MT15107	*lin-53(n3368)*	8,69 ± 0,16	vs. wt < 0,0001

Lifespan without FUDR

Strain	Genotype	Mean survival ± SEM in days	p-value (Log-Rank test)
N2	*wt*	12,31 ± 0,23	
MT15107	*lin-53(n3368)*	8,08 ± 0,18	vs. wt < 0,0001

Figure S2

Müthel et al.

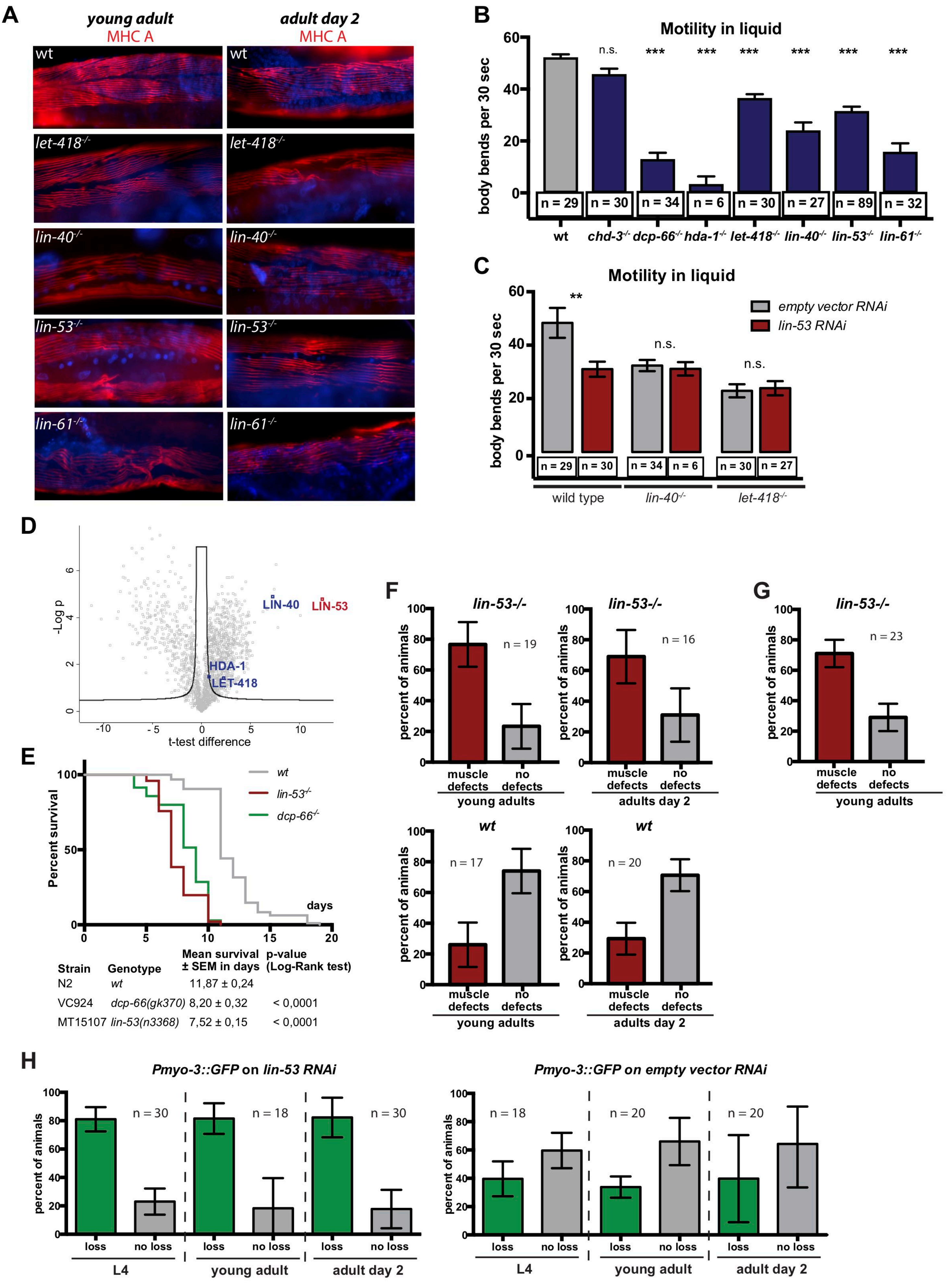

Figure S3

Müthel et al.

A

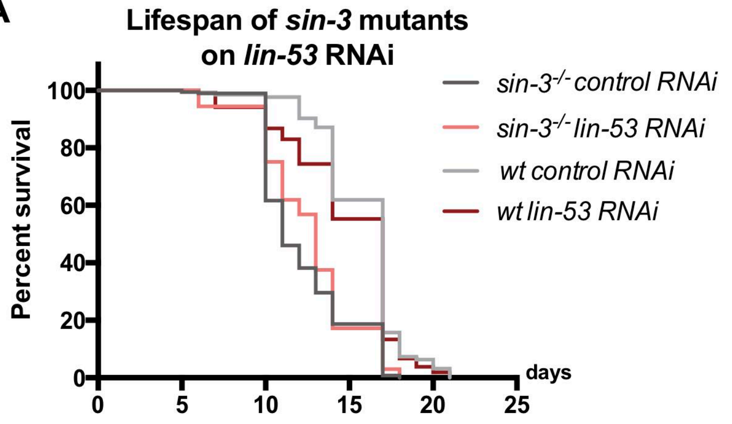

Strain	Genotype	RNAi	Mean survival ± SEM in days	p-value vs. control (Log-Rank test)
KC565	*sin-3(tm1276)*	*control*	12,28 ± 0,22	
KC565	*sin-3(tm1276)*	*lin-53*	12,64 ± 0,26	0,0919
N2	*wt*	*control*	16,24 ± 0,19	
N2	*wt*	*lin-53*	15,19 ± 0,23	0,0092

B

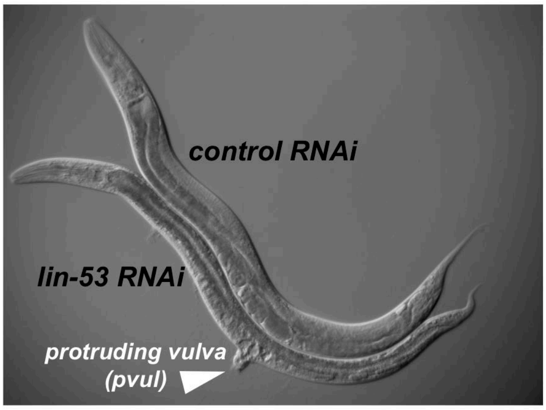

C

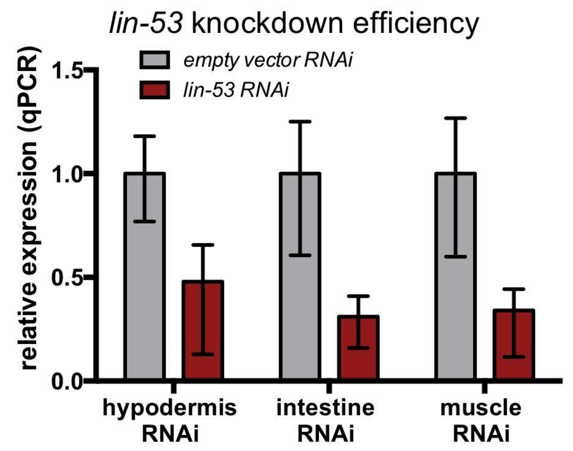

D

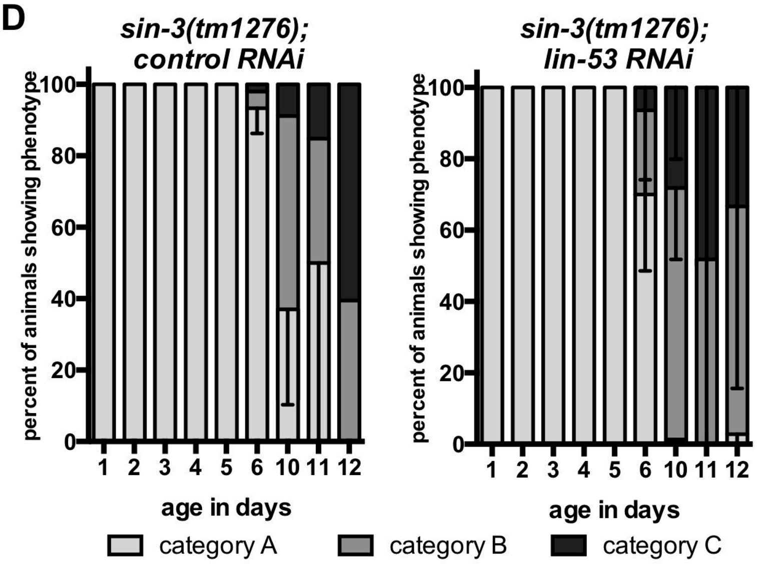

Figure S4 **Seelk-Müthel et al.**

A

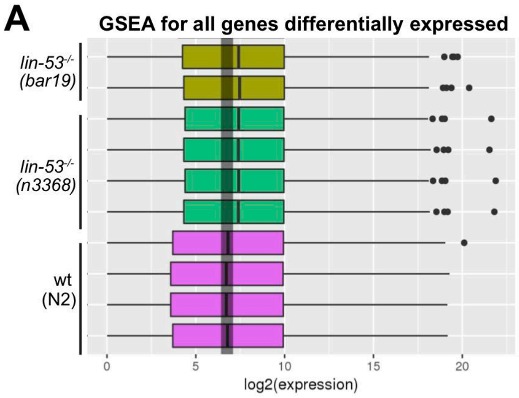

B

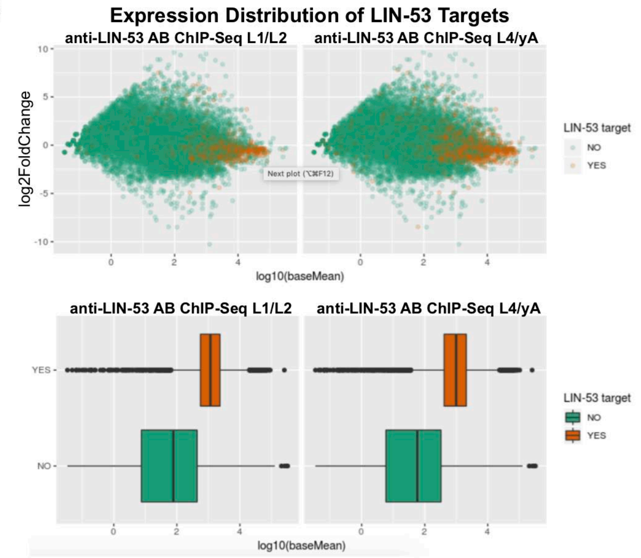

C

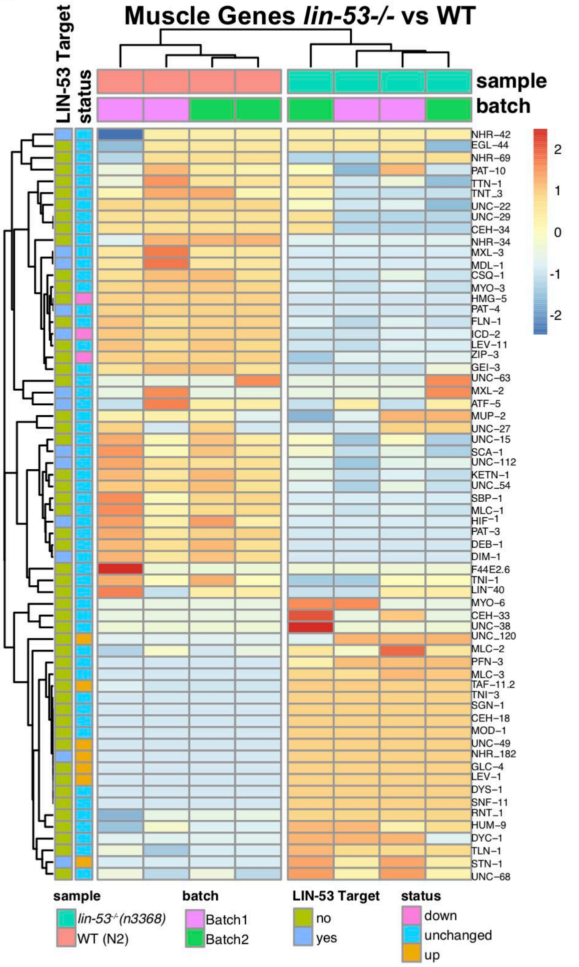

D

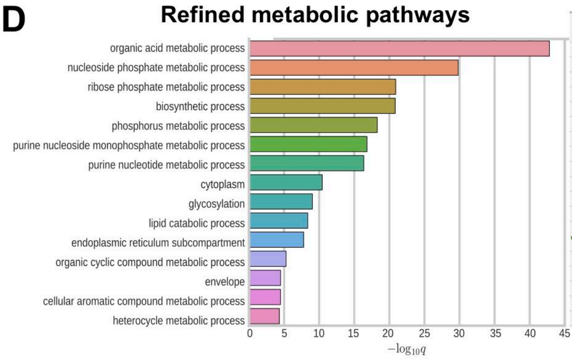

Expected	Observed	Enrich. FC	P value	Q value
12	84	7	1.2e-45	1.5e-43
8	57	7.1	2.4e-32	1.5e-30
6.3	42	6.7	2.9e-23	1.2e-21
57	142	2.5	4.6e-23	1.5e-21
35	99	2.8	2.1e-20	5.3e-19
3.4	28	8.2	7E-19	1.5e-17
5.8	35	6	2.4e-18	4.4e-17
1e+02	172	1.7	2.3e-12	3.7e-11
3.9	21	5.4	6.5e-11	9.2e-10
3.1	18	5.8	3.3e-10	4.3e-09
6.8	26	3.8	1.4e-09	1.6e-08
59	98	1.7	4.8e-07	5.1e-06
9.3	25	2.7	3.2e-06	3.1e-05
58	93	1.6	3.5e-06	3.2e-05
57	92	1.6	5.5e-06	4.7e-05
36	64	1.8	6.5e-06	5.2e-05
1.6e+02	196	1.2	0.0011	0.008
2.8	7	2.5	0.0076	0.054
45	60	1.3	0.013	0.09

E

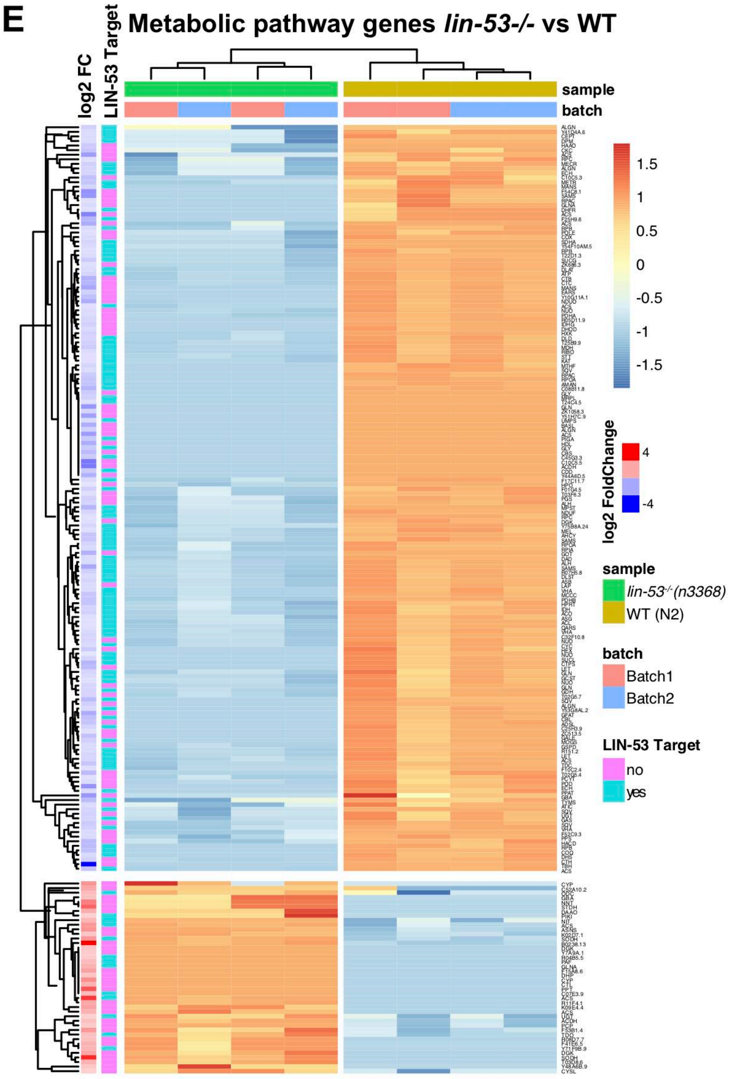

Figure S5 **Müthel et al.**

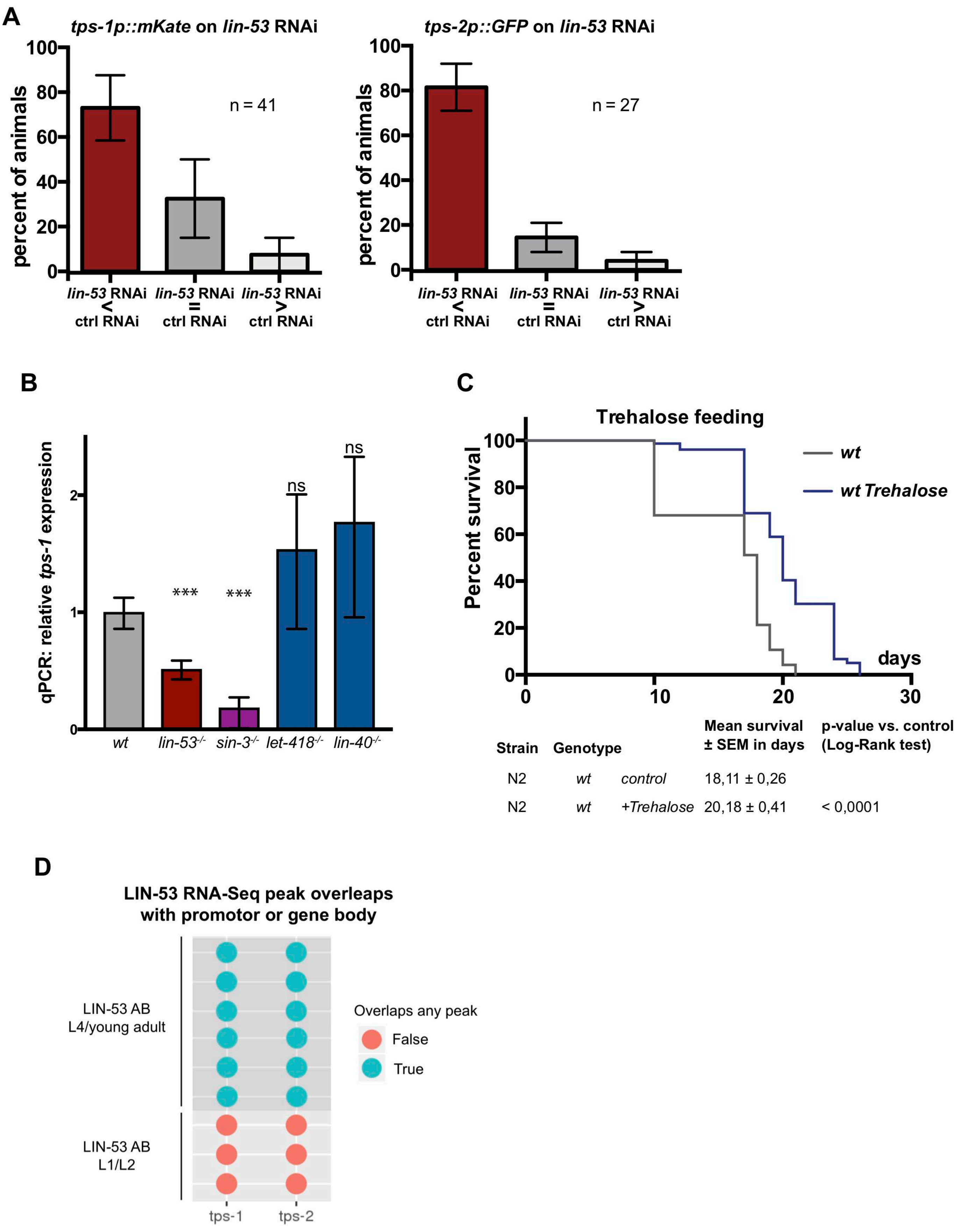

Figure S6

Müthel et al.

A

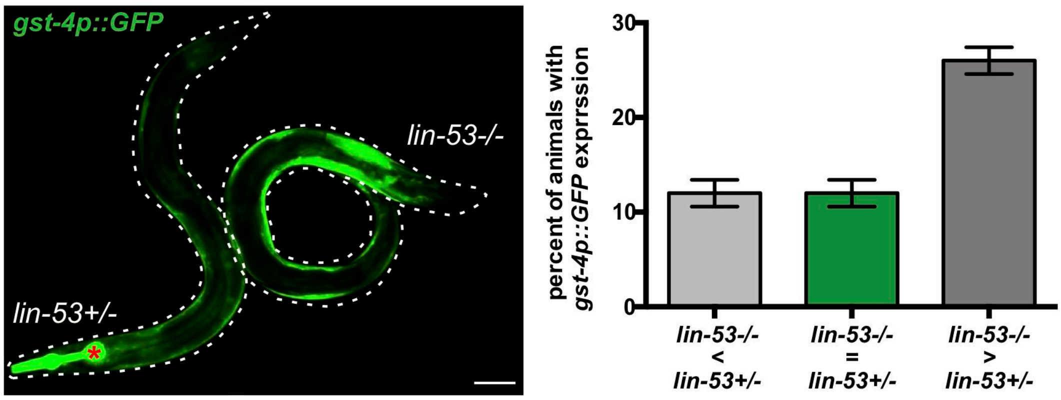

B

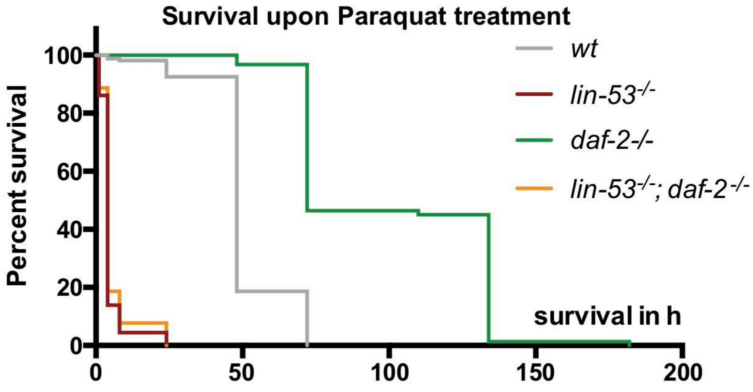

Strain	Genotype	treatment	Mean survival ± SEM in hours	p-value vs. control (Log-Rank test)
N2	*wt*	*Paraquat*	52,27 ± 1,18	
MT15107	*lin-53(n3368)*	*Paraquat*	4,66 ± 0,29	< 0,0001
CB1370	*daf-2(e1370)*	*Paraquat*	100,31 ± 2,66	< 0,0001
BAT376	*daf-2(e1370); lin-53(n3368)*	*Paraquat*	5,65 ± 0,56	vs. wt < 0,001<br>vs. daf-2-/- < 0,0001<br>vs. lin-53-/- < 0,6835

C

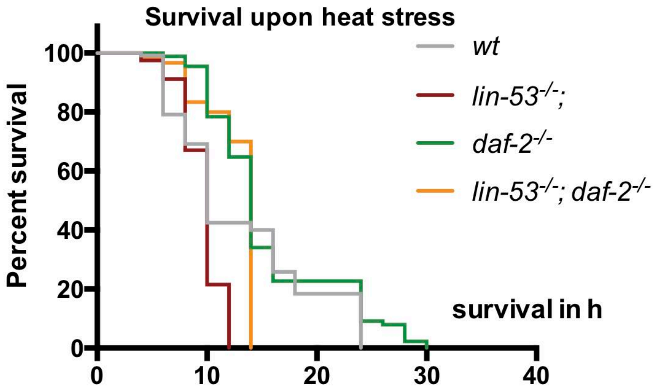

Strain	Genotype	treatment	Mean survival ± SEM in hours	p-value vs. control (Log-Rank test)
N2	*wt*	*37°C*	13,07 ± 0,58	
MT15107	*lin-53(n3368)*	*37°C*	9,48 ± 0,22	< 0,0001
CB1370	*daf-2(e1370)*	*37°C*	15,56 ± 0,62	0,0519
BAT376	*daf-2(e1370); lin-53(n3368)*	*37°C*	12,57 ± 0,33	vs. wt 0,4671<br>vs. daf-2-/- 0,0251<br>vs. lin-53-/- < 0,0001
