# Supplementary material for: The conserved histone chaperone LIN‐53 is required for normal lifespan and maintenance of muscle integrity in Caenorhabditis elegans
Source: Aging Cell. 2019 Aug 9;18(6):e13012. doi: 10.1111/acel.13012 (PMC6826145; doi:10.1111/acel.13012)
Supplement: Supplementary file 2 [file ACEL-18-e13012-s002.pdf]

Table S1, Müthel et al.

Strain	Genotype	Mean survival ± SEM in days	p-value vs. control (Log-Rank test)
N2	*wt*	13,66 ± 0,26	
MT15107	*lin-53(n3368)*	8,69 ± 0,16	< 0,0001

Strain	Genotype	Mean survival ± SEM in days	p-value vs. control (Log-Rank test)
N2	*wt*	9,64 ± 0,29	
MT15107	*lin-53(n3368)*	5,39 ± 0,17	
BAT1883	*baf-1p::GFP::lin-53::2xFLAG*	10,36 ± 0,70	vs. wt 0,2488
BAT729	*lin-53(n3368); myo-3p::lin-53::2xFLAG*	4,67 ± 0,12	vs. *lin-53*-/- 0,0002

Strain	Genotype	Mean survival ± SEM in days	p-value vs. control (Log-Rank test)
N2	*wt*	13,66 ± 0,26	
MT15107	*lin-53(n3368)*	8,69 ± 0,16	< 0,0001
BAT320	*lin-53 CRISPR*	9,12 ± 0,14	< 0,0001

Strain	Genotype	Mean survival ± SEM in days	p-value vs. control (Log-Rank test)
N2	*wt*	11,87 ± 0,24	
HX103	*chd-3(eh4)*	15,33 ± 0,30	< 0,0001
MT14390	*let-418(n3536)*	15,66 ± 0,18	< 0,0001
VC660	*lin-40(ok905)*	11,20 ± 0,33	0,0796
MT15107	*lin-53(n3368)*	7,52 ± 0,15	< 0,0001
MT12833	*lin-61(n3809)*	15,43 ± 0,20	< 0,0001

Strain	Genotype	Mean survival ± SEM in days	p-value vs. control (Log-Rank test)
N2	*wt*	13,66 ± 0,26	
BAT320	*lin-53(bar19)*	9,12 ± 0,14	< 0,0001

Strain	Genotype	Mean survival ± SEM in days	p-value vs. control (Log-Rank test)
N2	*wt*	11,87 ± 0,24	
MT15107	*lin-53(n3368)*	8,69 ± 0,16	< 0,0001
KC565	*sin-3(tm1276)*	10,46 ± 0,15	< 0,0001

Strain	Genotype	Mean survival ± SEM in days	p-value vs. control (Log-Rank test)
N2	*wt*	11,87 ± 0,24	
MT15795	*isw-1(n3294)*	15,16 ± 0,27	< 0,0001
MT13649	*nurf-1(n4295)*	17,62 ± 0,27	< 0,0001

Strain	Genotype	Mean survival ± SEM in days	p-value vs. control (Log-Rank test)
N2	*wt*	11,87 ± 0,24	
MT20434	*chaf-1(n5453)*	14,76 ± 0,30	< 0,0001

Strain	Genotype	RNAi	Mean survival ± SEM in days	p-value vs. control (Log-Rank test)
N2	*wt*	*control*	16,24 ± 0,19	
N2	*wt*	*lin-53*	15,19 ± 0,23	0,0092

Strain	Genotype	treatment	Mean survival ± SEM in hours	p-value vs. control (Log-Rank test)
N2	*wt*	*37°C*	13,07 ± 0,58	
MT15107	*lin-53(n3368)*	*37°C*	9,48 ± 0,22	< 0,0001
CB1370	*daf-2(e1370)*	*37°C*	15,56 ± 0,62	0,0519
BAT376	*daf-2(e1370); lin-53(n3368)*	*37°C*	12,57 ± 0,33	vs. wt 0,4671<br>vs. daf-2-/- 0,0251<br>vs. lin-53-/- < 0,0001

Strain	Genotype	RNAi	Mean survival ± SEM in days	p-value vs. control (Log-Rank test)
KC565	*sin-3(tm1276)*	*control*	12,28 ± 0,22	
KC565	*sin-3(tm1276)*	*lin-53*	12,64 ± 0,26	0,0919
N2	*wt*	*control*	16,24 ± 0,19	
N2	*wt*	*lin-53*	15,19 ± 0,23	0,0092

Strain	Genotype	treatment	Mean survival ± SEM in hours	p-value vs. control (Log-Rank test)
N2	*wt*	*Paraquat*	52,27 ± 1,18	
MT15107	*lin-53(n3368)*	*Paraquat*	4,66 ± 0,29	< 0,0001
CB1370	*daf-2(e1370)*	*Paraquat*	100,31 ± 2,66	< 0,0001
BAT376	*daf-2(e1370); lin-53(n3368)*	*Paraquat*	5,65 ± 0,56	vs. daf-2-/- < 0,0001<br>vs. lin-53-/- < 0,6835

Strain	Genotype	survival ± SEM in	p-value vs. control (Log-Rank test)
N2	*wt*	9,64 ± 0,29	
MT15107	*lin-53(n3368)*	5,39 ± 0,17	
BAT1883	*lin-53(n3368); barEx974 [baf-1p::GFP::lin-53::2xFLAG]*	10,36 ± 0,7	vs. lin-53-/- < 0,0001<br>vs. wt 0,2488
	lin-53(n3368); barIS87 [myo-3p::lin-53::2xFLAG]	4,67 ± 0,12	vs. lin-53-/- 0,0002
BAT729	*barIS87 [myo-3p::lin-53::2xFL*	8,94 ± 0,29	vs. wt 0,0047
BAT1943	*barSi23 [baf-1p::GFP::lin-53::2xFLAG:: SL2::NLS::tagRFP]*	11,64 ± 0,1	vs. wt < 0,0001

Strain	Genotype	Mean survival ± SEM in days	p-value vs. control (Log-Rank test)
N2	*wt*	11,87 ± 0,24	
VC764	*hat-1(ok1265)*	16,93 ± 0,34	< 0,0001

Strain	Genotype	Mean survival ± SEM in days	p-value vs. control (Log-Rank test)
N2	*wt*	11,87 ± 0,24	
MT8839	*lin-52(n771)*	19,43 ± 0,30	< 0,0001
MT5470	*lin-37(n758)*	14,66 ± 0,24	< 0,0001
MT11147	*dpl-1(n3643)*	16,74 ± 0,28	< 0,0001

Strain	Genotype	Mean survival ± SEM in days	p-value vs. control (Log-Rank test)
N2	*wt*	13,66 ± 0,26	
MT15107	*lin-53(n3368)*	8,69 ± 0,16	< 0,0001
CB1370	*daf-2(e1370)*	28,29 ± 0,75	< 0,0001
BAT376	*daf-2(e1370); lin-53(n3368)*	14,02 ± 0,47	vs. wt 0,2954<br>vs. daf-2-/- < 0,0001<br>vs. lin-53-/- < 0,0001

Strain	Genotype	Mean survival ± SEM in days	p-value vs. control (Log-Rank test)
N2	*wt*	13,66 ± 0,26	
MT15107	*lin-53(n3368)*	8,69 ± 0,16	< 0,0001
CB1370	*daf-2(e1370)*	28,29 ± 0,75	< 0,0001
BAT762	*daf-2(e1370); sin-3(tm1276)*	22,42 ± 0,63	vs. wt < 0,0001<br>vs. daf-2-/- < 0,0001<br>vs. lin-53-/- < 0,0001

Strain	Genotype		Mean survival ± SEM in days	p-value vs. control (Log-Rank test)
MT15107	*lin-53(n3368)*	*control*	9,78 ± 0,14	
MT15107	*lin-53(n3368)*	*Trehalose*	10,88 ± 0,17	< 0,0001
KC565	*sin-3(tm1276)*	*control*	10,46 ± 0,15	
KC565	*sin-3(tm1276)*	*Trehalose*	12,34 ± 0,28	< 0,0001
N2	*wt*		13,66 ± 0,26	
N2	*wt*	*Trehalose*	20,18 ± 0,41	< 0,0001

Strain	Genotype		Mean survival ± SEM in days	p-value vs. control (Log-Rank test)
N2	*wt*	*control*	18,11 ± 0,26	
N2	*wt*	*Trehalose*	20,18 ± 0,41	< 0,0001

Lifespan Data Figure S1F

Repeat I

**%BAT1943**		
#day	dead	censored
0	0	0
1	0	0
2	0	0
3	0	0
4	0	0
7	0	0
8	0	0
9	0	0
10	0	0
14	1	0
15	0	0
16	1	0
17	8	0
21	16	0
22	2	1
23	4	0
24	5	0
28	10	0
29	2	0

Repeat II

**%BAT1943**		
#day	dead	censored
0	0	0
1	0	0
2	0	0
3	0	0
4	0	0
7	0	0
8	0	1
9	0	0
10	0	0
14	2	0
15	2	1
16	1	0
17	0	1
21	10	10
22	4	0
23	7	0
24	3	0
28	4	0
29	0	0
30	0	0
31	0	0
32	1	0
35	3	0

Repeat III

**%BAT1943**		
#day	dead	censored
0	0	0
1	0	0
2	0	0
3	0	0
4	0	0
7	0	0
8	0	0
9	0	0
10	0	0
14	3	0
15	2	0
16	3	0
17	8	0
21	13	0
22	5	0
23	3	0
24	3	0
28	10	0

Repeat I

**%N2**		
#day	dead	censored
0	0	0
1	0	0
2	0	0
3	0	0
4	0	0
7	0	0
8	0	0
9	0	0
10	0	0
14	2	1
15	1	0
16	3	0
17	10	2
21	14	1
22	8	0
23	6	0
24	0	0
28	2	0

Repeat II

**%N2**		
#day	dead	censored
0	0	0
1	0	0
2	0	0
3	0	0
4	0	0
7	0	4
8	0	0
9	0	0
10	0	0
14	2	0
15	1	1
16	4	0
17	18	0
21	3	4
22	3	0
23	4	0
24	3	0
28	3	0

Repeat III

**%N2**		
#day	dead	censored
0	0	0
1	0	0
2	0	0
3	0	0
4	0	0
7	0	2
8	0	0
9	0	2
10	0	1
14	8	6
15	2	0
16	2	0
17	10	0
21	10	0
22	2	0
23	1	0
24	2	0
28	2	0

%BAT1943		
#day	dead	censored
0	0	0
1	0	0
2	0	0
3	0	0
4	0	0
7	0	0
8	0	1
9	0	0
10	0	0
14	6	0
15	4	1
16	5	0
17	16	1
21	39	10
22	11	1
23	14	0
24	11	0
28	24	0
29	2	0
30	0	0
31	0	0
32	1	0
35	3	0

%N2		
#day	dead	censored
0	0	0
1	0	0
2	0	0
3	0	0
4	0	0
7	0	6
8	0	0
9	0	2
10	0	1
14	12	7
15	4	1
16	9	0
17	38	2
21	27	5
22	13	0
23	11	0
24	5	0
28	7	0

Strain	Genotype	RNAi	Mean survival ± SEM in days	Number of animals died/total	p-value vs. control (Log-Rank test)	remark
N2	*wt*		13,66 ± 0,26	130/150		
MT15107	*lin-53(n3368)*		8,69 ± 0,16	174/174	< 0,0001	
CB1370	*daf-2(e1370)*		28,29 ± 0,75	128/150	< 0,0001	
BAT376	*daf-2(e1370); lin-53(n3368)*		14,02 ± 0,47	104/118	vs. wt 0,2954<br>vs. daf-2-/- < 0,0001<br>vs. lin-53-/- < 0,0001	
BAT320	*lin-53(bar19)*		9,12 ± 0,14	133/150	< 0,0001	
N2	*wt*		9,64 ± 0,29	127/150		animals were kept continiously at 25°C; scoring was started at L4 stage
MT15107	*lin-53(n3368)*		5,39 ± 0,17	128/140		
BAT1883	*lin-53(n3368); barEx974 [baf-1p::GFP::lin-53::2xFLAG]*		10,36 ± 0,70	19/25	vs. lin-53-/- < 0,0001<br>vs. wt 0,2488	
	lin-53(n3368); barIS87 [myo-3p::lin-53::2xFLAG]		4,67 ± 0,12	132/140	vs. lin-53-/- 0,0002	
BAT729	*barIS87 [myo-3p::lin-53::2xFLAG]*		8,94 ± 0,29	94/150	vs. wt 0,0047	
BAT1943	*barSi23 [baf-1p::GFP::lin-53::2xFLAG::SL2::NLS::tagRFP]*		11,64 ± 0,16	194/200	vs. wt < 0,00001	
N2	*wt*		11,87 ± 0,24	95/95		animals were kept continiously at 25°C
HX103	*chd-3(eh4)*		15,33 ± 0,30	129/139	< 0,0001	
VC924	*dcp-66(gk370)*		8,20 ± 0,32	35/35	< 0,0001	
MT14390	*let-418(n3536)*		15,66 ± 0,18	213/213	< 0,0001	
VC660	*lin-40(ok905)*		11,20 ± 0,33	75/75	0,0796	
MT15107	*lin-53(n3368)*		7,52 ± 0,15	122/122	< 0,0001	
MT12833	*lin-61(n3809)*		15,43 ± 0,20	134/150	< 0,0001	
MT15795	*isw-1(n3294)*		15,16 ± 0,27	140/150	< 0,0001	
MT13649	*nurf-1(n4295)*		17,62 ± 0,27	132/150	< 0,0001	
MT8839	*lin-52(n771)*		19,43 ± 0,30	140/150	< 0,0001	
MT5470	*lin-37(n758)*		14,66 ± 0,24	121/150	< 0,0001	
MT11147	*dpl-1(n3643)*		16,74 ± 0,28	123/150	< 0,0001	
MT20434	*chaf-1(n5453)*		14,76 ± 0,30	140/145	< 0,0001	
KC565	*sin-3(tm1276)*		10,46 ± 0,15	108/130	< 0,0001	
VC764	*hat-1(ok1265)*		16,93 ± 0,34	128/150	< 0,0001	
N2	*wt*		13,66 ± 0,26	130/150		
CB1370	*daf-2(e1370)*		28,29 ± 0,75	128/150	< 0,0001	
KC565	*sin-3(tm1276)*		10,46 ± 0,15	108/130	< 0,0001	
BAT762	*daf-2(e1370); sin-3(tm1276)*		22,42 ± 0,63	131/148	vs. wt < 0,0001<br>vs. daf-2-/- < 0,0001<br>vs. lin-53-/- < 0,0001	
KC565	*sin-3(tm1276)*	*control*	12,28 ± 0,22	115/150		
KC565	*sin-3(tm1276)*	*lin-53*	12,64 ± 0,26	110/145	0,0919	
N2	*wt*	*control*	18,11 ± 0,26	36/50	< 0,0001	
N2	*wt*	*Trehalose*	20,18 ± 0,41	61/94	< 0,0001	
MT15107	*lin-53(n3368)*	*control*	9,78 ± 0,14	242/250		
MT15107	*lin-53(n3368)*	*Trehalose*	10,88 ± 0,17	11/145	< 0,0001	
KC565	*sin-3(tm1276)*	*control*	10,46 ± 0,15	108/130		
KC565	*sin-3(tm1276)*	*Trehalose*	12,34 ± 0,28	137/145	< 0,0001	
N2	*wt*	*control*	16,24 ± 0,19	120/175		
N2	*wt*	*lin-53*	15,19 ± 0,23	163/200	0,0092	
N2	*wt*	*37°C*	13,07 ± 0,58	120/120		
MT15107	*lin-53(n3368)*	*37°C*	9,48 ± 0,22	80/80	< 0,0001	
CB1370	*daf-2(e1370)*	*37°C*	15,56 ± 0,62	90/90	0,0519	
BAT376	*daf-2(e1370); lin-53(n3368)*	*37°C*	12,57 ± 0,33	60/60	vs. wt 0,4671<br>vs. daf-2-/- 0,0251<br>vs. lin-53-/- < 0,0001	
N2	*wt*	*control*	18,11 ± 0,26	36/50		
N2	*wt*	*Trehalose*	20,18 ± 0,41	62/95	< 0,0001	
