# Supplementary material for: The conserved histone chaperone LIN‐53 is required for normal lifespan and maintenance of muscle integrity in Caenorhabditis elegans
Source: Aging Cell. 2019 Aug 9;18(6):e13012. doi: 10.1111/acel.13012 (PMC6826145; doi:10.1111/acel.13012)
Supplement: Supplementary file 3 [file ACEL-18-e13012-s003.pdf]

Table S2, Müthel et al.

Gene names of proteins identified by IP-MS using *myo-3p::lin-5::2xFLAG*
pck-2 rop-1 pah-1 gpdh-2 gdi-1 tpi-1 pgp-2 F18E3.7 nxf-1 lin-40 tsn-1 mccc-1 cct-1 idh-2 pccb-1 elpc-3 dyci-1 ogt-1 elo-1 phy-2 cts-1 tufm-2 ugt-46 dpy-23 ppm-1 pfk-1 T01C8.5 pcca-1 cct-6 ZK1073.1 rpt-4 unc-78 ben-1 cct-8 pab-2 pyc-1 F14E5.2 Y39E4A.3 ZK686.3 hat-1 got-2.2 cct-4 uba-1 gpi-1 pdi-1 gst-7 gpb-2 cct-2 mdh-1 sur-6 pme-5 npp-20 unc-116 mec-7 aco-2 rpt-3 hgrs-1 pmp-4 cct-7 unc-45 Y57G11C.3 cpt-2 sac-1 tbb-2 nsf-1 rpt-6 cct-5 sec-23 smc-3 dnj-12 pbs-3 F02A9.4 pgk-1 ufd-3 tax-6 F46H5.3 rpt-1 T09A5.11 let-721 acp-6 upb-1 pdi-2 aak-2 cct-3 gpx-2 lin-53 gdh-1 rpl-31 lis-1 tps-1 F38A5.1 C48B4.1 gst-5 rpt-2gsy-1 4D656 gta-1 unc-60 alh-3 gei-7;icl-1 F21H12.6 dnj-19 rpoa-1 dis-3 alh-13 dhs-12 lec-6 asp-3 pbs-4 cri-3 upp-1 fce-1 pam-1 acdh-11 hpo-29 gst-27 aco-1 rpl-18 aagr-4 rpb-2 dpf-5 unc-61 R05D3.9/R05D3.10 smk-1 rpc-1 ekl-1 glrx-3 csn-4 sod-2 pph-5 nduf-7 tufm-1 cyn-5 apa-2 emb-4 rpl-26 gpd-2 aly-1 rpoa-2 rpl-13 fmo-1 cox-2 lars-2 plrg-1 acs-22 rpl-23 nud-1 nuo-4 fnta-1 srp-7 rpl-6 ppw-1 C05G5.4 iars-2 dlst-1 alg-1 Y38F1A.6 abce-1 gsr-1 C05D11.1 bub-3 rpl-17 rpl-10 C28H8.3 dnj-20 lin-23 tba-1 rpl-43 skih-2 gsa-1 ama-1 rpb-5 ife-1 fzo-1 ubq-2;ubq-1 dsc-4 mrps-9 rpc-2 sur-5 usp-5 Y94H6A.8 dhc-1 B0303.3 rars-1;rrt-1 rpn-3 gyg-1 cor-1 dpy-18 vpr-1 let-92 C30C11.4 rps-21 emb-8 fum-1 hpd-1 C10C6.6 cul-2 dld-1 dpy-11 ZK180.4 C05C10.3 acdh-9 rps-25 hsp-4 anc-1 rpb-11 dif-1 nuo-5 tag-210 snt-1 rpn-9 elpc-1 nduf-6 B0250.5 csn-2 pmk-3 math-33; usp-7 apy-1 rack-1 din-1 nuo-6 rpn-5 gst-20 eat-3 clp-1 F38B2.4 ostd-1 ile-1 idha-1 tkt-1 gale-1 faah-1 C06G3.9 ZK370.8 pptr-1 frs-2 F38E11.5 aldo-1 rskn-2 drs-1 idhb-1 H24K24.3 pyk-2 dct-18 aldo-2 ccdc-47 cey-1 unc-32 srs-2 T22D1.3 glt-1 K12H4.5 rla-1 vha-15 dnj-11 byn-1 eif-3.B gly-4 irs-1 dli-1 nuo-1 gst-13 daf-21 ragc-1 spg-7 R07B7.5 haf-9 alh-1 cash-1 cyn-3 F32D1.5 mca-1 cbs-1 ech-6 acdh-12 dpt-1 alx-1 let-711 vrs-2 asg-2 hsp-1 tag-320 tag-335 alh-7 mmcm-1 H28O16.1 hsp-6 pyp-1 enpl-1 abtm-1 cars-1 par-5 B0513.5 B0286.3 dhs-28 acs-19 rpt-5 atgp-2 pfn-3 dpyd-1 vha-5 C34B2.6 car-1 rme-1 F47B10.1 clu-1 T20B12.7 cand-1 rme-8 enol-1 fbp-1 cey-4 rpn-1 F55A12.8 cul-1 eif-3.L ife-2 asb-2 txl lap-1 hsp-3 C04C3.3 rpn-12 dyn-1 eif-6 cdr-4 sec-24.1 alh-4 pfn-1 eef-2 K02D10.1 T14G10.5 rpn-10 sfxn-5 lrp-1 ruvb-2 T03F1.1 let-418 pas-5 Y45G12B.3 oig-2 sec-31 rps-10 prdx-2 ctl-2 C37H5.6 T22B11.5 spl-1 C16A3.10 agl-1 Y113G7B.16 ima-3 rpb-3 mdh-2 acdh-10 gop-3 set-18 C18A3.3 F23B12.5 cogc-4 crt-1 cysl-2 lst-3 eif-3.G;eif-3.g lec-9 vha-13 app-1 nkb-3 hsp-60 copb-1 rpl-11.1 K12H4.4 C13B9.3 gst-26 imb-1 ivd-1 unc-43 pars-1 alh-8 puf-9 prdx-3 rpn-6.1 egl-4 C52E4.5 ZK550.6 drp-1 immt-1 F52C9.3 tim-44 chc-1 rpn-7 vha-11 lrs-1 ech-5 eat-6 mca-2 alh-6 tbb-1 dnj-16 cdc-48.2 F37C4.5 sip-1 C56G2.7 acs-2 tba-4 mct-6 snr-1 mec-12 nnt-1 pbs-1 set-27 sodh-1 tag-131 atgp-1 tba-7 pas-3 npp-17 pck-1 acdh-7 unc-22 pud-1.2 gars-1;grs-1 pbs-5 Y37D8A.10 cdc-48.1 xrn-1 dig-1 prp-19 pud-3 glrx-5 fib-1 pas-7 sdha-1 sel-1 asp-5 sft-4 tat-4 pyk-1 poml-3;poml-4 teg-4 usp-14 gpd-4;gpd-1 cpr-4
