# Supplementary material for: The conserved histone chaperone LIN‐53 is required for normal lifespan and maintenance of muscle integrity in Caenorhabditis elegans
Source: Aging Cell. 2019 Aug 9;18(6):e13012. doi: 10.1111/acel.13012 (PMC6826145; doi:10.1111/acel.13012)
Supplement: Supplementary file 4 [file ACEL-18-e13012-s004.pdf]

Table S3, Müthel et al.,

	wt	*daf-2*$^{-/-}$	*lin-53*$^{-/-}$	*lin-53*$^{-/-}$; [illegible]	*sin-3*$^{-/-}$	*let-418*$^{-/-}$
2-Piperidinecarboxylic acid	0	0	0	0	-0,002671	0,75
Acetoacetic acid	-0,620735	-0,006544	1,836014	0,5468859	-0,580722	9,069182
Adenine	0,1965731	0,1353308	-0,729864	-1,241531	-0,705337	0,5801718
Adenosine	-4,011155	-12,1066	-0,011794	0,907314	-0,01004	0,6673211
Alanine_ beta-	0,5142059	-1,39436	-12,33701	-7,28596	10,82302	-3,237265
Alanine_ beta	36,14277	-5,630155	-7,795528	-4,021849	3,02437	-3,440542
Alanine_ N-acetyl-_ DL-	-1,329262	0,7199413	-0,020311	-0,020311	-0,001999	-0,001999
Alanine	-0,330026	-0,699524	1,100601	0,3458593	-2,364466	0,0882185
Arginine	-0,294054	-0,279054	0,5108386	1,728811	0,3463029	-0,461224
Ascorbic acid	-0,009514	0,75	-0,009514	-0,009514	0,75	-0,019135
Asparagine	-0,318912	0,5182338	-0,937319	-0,538637	-1,764087	-19,76633
Aspartic acid	-0,198795	-0,568179	4,793085	0,130141	-1,587684	0,2993085
Benzoic acid	-2,717209	0,2544456	2,711601	1,656488	0,713729	-1,491187
Butanoic acid_ 2-amino-	0,3685384	-4,474225	1,7674	-4,408711	6,314149	-4,279081
Butanoic acid_3-hydroxy	0,5406324	-7,654907	-0,828941	4,74594	2,700768	-1,347472
Butanoic acid_4-amino	-0,578621	-0,425403	0,7574725	0,4733723	-0,145136	-1,709611
Butanoic acid_ 4-hydroxy-	-0,739114	0,5942206	3,122558	-0,116453	-0,642391	3,954789
Citric acid	-0,419723	-109,251	-15,21331	3,863712	-7,162234	0,6612802
Cystathionine	-0,162676	0,4662593	-3,418903	0,2479245	-0,209538	-5,637393
Cysteine	-8,270255	2,274708	-40,25044	0,6543203	-0,039634	0,4409374
Cysteinesulfinic acid	-0,366209	-12,24777	0,9596263	0,0327225	1,071155	-0,947606
Cytosine	0,1626125	0,2588254	-0,391397	-0,589162	-0,12742	-1,913104
Dehydroascorbic acid dimer	-1,25308	6,53667	0,0323061	-0,009163	4,294538	0,2463672
Erythritol	-0,61971	-0,309073	0,289463	0,2796391	3,361274	-0,518662
Erythrose-4-phosphate	-0,040298	1,04161	-0,664433	1,029509	1,741889	-0,343954
Ethanolamine	0,1969122	-1,010669	0,2782534	-1,407194	-0,523337	-8,000259
Ethanolaminephosphate	-0,020831	0,60517	-0,594999	-0,190045	-0,046883	0,1992403
Fructose	-0,75023	0,0955214	1,333688	1,618862	1,92906	-3,917138
Fructose-1_6-diphosphate	1,036032	0,7032653	-1,217174	-1,600726	1,735205	-2,352188
Fructose-1-phosphate	0,2575838	1,169619	-0,00639	-0,019589	0,9534482	-3,161124
Fructose-6-phosphate	0,0784286	1,196019	-3,374202	-3,513191	1,790589	-0,966569
Fumaric acid	-0,398153	-1,680027	-0,734614	1,089881	-0,114158	0,7898067
Galactitol	0,0304638	-0,015898	-0,015898	5,641377	1,938481	4,866229
Galactosamine_ N-acetyl-	-1,346544	-15,07428	2,006886	1,343536	-3,098015	-200,9925
Galactose	-41,8918	-2,711452	2,393225	13,41705	0,4109716	-38,90169
Galacturonic acid	-0,340642	0,6249092	-0,318228	0,0540031	-0,396699	0,1376916
Gluconic acid	0,75	-0,003482	-0,003482	-0,003482	-0,00991	-0,00991
Gluconic acid-6-phosphate	0,497642	-2,825186	2,505224	-1,277572	0,0264505	7,87739
Glucopyranose	-0,002194	0,3473559	0,5947761	-0,002194	0	0
Glucosamine_ N-acetyl-	0	0	0	0	-0,006417	-0,006417
Glucosamine_ N-acetyl-	-0,001465	0,5482323	-0,001465	0,4402379	-1,456277	-2,183697
Glucosamine	-0,128914	2,738886	-0,017746	-1,711153	2,115628	3,82472
Glucose_ 2-deoxy-	-0,54384	2,627323	-0,203527	1,734852	7,180919	-2,186672
Glucose	0,2250236	2,070801	-1,125935	-0,369312	-0,042396	1,156248
Glucose-1/6-phosphate	0,0898783	0,6472971	-3,751423	11,3332	1,698237	-1,223157
Glucose-6-phosphate_ 2-deoxy-	0	0	0	0	0	0
Glucose-6-phosphate	0,3886879	1,757779	-142,3394	-75,8309	2,073034	-4,284975
Glucuronic acid	0,3824831	0,7699584	-1,212465	-7,598788	2,582741	0,6476094
Glutamic acid_ N-acetyl-	0	0	0	0	-0,002082	-0,002082
Glutamic acid	0,2574993	-0,13543	-1,572243	0,0792657	0,7425779	19,17679
Glutamine	-0,309372	-0,450203	-284,0164	5,702925	-0,793866	0,5651649
Glutamine_ N-acetyl-	-4,559418	0,5569087	-0,060352	-2,692668	-3,190953	-1,354814
Glutamine	0,2125266	0,5039539	-1,381692	-2,397837	-0,128939	-20,16927
Glutaric acid_ 2-hydroxy-	0,2933148	1,427064	-66,25396	0,5092276	0,4657097	1,556717
Glutaric acid_ 2-oxo-	0,75	-0,001895	-0,001895	-0,001895	0	0
Glutaric acid	-1,251494	0,5134022	0,39242	-16,70929	3,6846	-4,813528
Glyceraldehyde	0,8279126	-5,022386	-3,006089	-7,790381	0,7494857	-110,3346
Glyceraldehyde-3-phosphate	0,5410064	-2,5592	-0,239939	-4,668027	1,166118	-8,587812

Glyceric acid	-1,090534	0,027869	0,477596	0,638524	0,7880896	-0,037982
Glyceric acid-2-phosphate	-0,293642	1,869878	-3,689701	7,026233	-0,291653	0,7544769
Glyceric acid-3-phosphate	-0,253333	1,268061	-13,3407	9,642215	-0,651339	0,3581929
Glycerol	-2,290356	0,831713	-0,027276	1,654048	0,1002426	-0,17274
Glycerol-2-phosphate	-0,286237	0,3057138	0,1829698	-2,948848	0,2936097	-1,410711
Glycerophosphoglycerol	-0,005481	-0,005481	-0,005481	1,288256	-0,009018	-0,009018
Glycine	-0,358017	1,116408	-5,358448	2,181579	1,643666	0,7064474
Glycolic acid	-0,039869	-7,043739	0,425081	0,512067	-3,847517	-0,305476
Guanosine	0	0	0	0	-0,018328	-0,018328
Guanosine-5-monophosphate	-0,013252	-0,013252	0,75	-0,013252	-0,032513	0,3509083
Gulonic acid	0,3105026	-0,234608	-0,920156	27,68282	8,644011	0,349885
Histamine	-0,066966	0,4073635	-0,274479	1,111439	-0,215027	-0,090914
Histidine	-0,000574	0,75	-0,000574	-0,000574	-0,083843	-1,728003
Homocysteine	0	0	0	0	0	0
Homoserine	-1,437046	-0,591702	-0,080761	1,704541	0,6891534	-1,932542
Hypotaurine	-0,121337	0,1508838	-0,757694	8,692596	0,0662358	-2,567103
Hypoxanthine	0,350359	0,9494863	-0,002786	-0,002786	-0,004535	0,3956108
Inosine	0	0	0	0	0	0
Inositol-2-phosphate	-0,000959	-0,000959	-0,000959	0,75	0	0
Isoascorbic acid	0,0344978	-0,059187	-0,944942	3,832192	-3,620577	0,8394344
Isobutanoic acid_ 3-amino-	0,5601285	-1,090022	-0,8814	-4,510427	0,8715749	-5,669307
Isoleucine	0,202468	-2,987519	0,8186974	-0,121223	9,020466	0,2496021
Isoleucine	-0,203608	-0,229494	3,283011	-1,316488	-0,233969	-6,256718
Lactic acid	0,5070343	0,1648594	-93,88633	-1,15682	-7,39125	3,353325
Lactose	-26,85761	0,7473726	-1,133459	-174,2921	-0,031458	-35,66237
Leucine	-0,463488	0,0689306	4,503948	-0,083904	0,1754178	0,7088981
Levulinic acid_ 5-amino-	-0,118595	-4,100986	-2,348786	2,254304	0,9420624	-0,001291
Lysine	0,2757433	-0,149819	-0,67873	-2,170933	23,14212	-1,40781
Maleic acid	-11,69536	0,9566368	-0,895582	0,7374615	0,0816847	1,907567
Malic acid	-0,228115	0,5769433	-12,50136	2,315668	0,087697	0,223721
Maltose	0,0617301	0,3215288	-0,728836	-1,356563	7,706681	0,4904831
Maltotriose	-0,663078	0,238653	0,3076684	0,0060451	-3,652486	-10,32056
Mannose	-0,075024	0,7328588	-2,099563	-3,176564	-4,570375	0,3939512
Methionine	-0,064417	-1,260909	30,26989	-4,635871	21,19572	-0,475204
Mevalonic acid-1_5-lactone	-0,236561	0,4484861	0,3400815	-4,813451	-2,943592	0,0752786
Neuraminic acid_ N-glycolyl-	-0,165295	0,5448008	-0,165295	12,99763	0,4197398	0,5607553
Nicotinamide	-0,684119	1,032014	-0,391059	0,7661854	2,479509	21,57335
Nicotine	-2,28423	-0,098265	0,4326876	0,0218044	-1,22037	0,0709916
Nonenal_ 4-hydroxy	-1,0264	-5,182608	0,7288355	-2,058349	-2,93975	-0,031518
Noradrenaline	-0,002171	0,6014058	-0,002171	0,3293914	-0,002948	-0,002948
Ornithine	0,4194494	-0,554424	-3,212446	-0,345211	-0,557554	-1,671726
Oxalic acid	0,374424	0,665133	-0,012531	0,383151	1,018106	-12,19571
Pantothenic acid	-0,633493	0,9341186	-1,063563	33,80168	2,158857	-1,613933
Phenylalanine	-0,29112	-1,570057	1,784727	-0,628695	3,073978	3,656655
Phosphoenolpyruvic acid	0	0	0	0	-0,013914	0,75
Phosphoric acid monomethyl ester	-1,082862	-5,04782	31,86477	3,236883	8,511449	-4,027376
Proline_ 4-hydroxy-_ trans-	-0,008511	0,75	-0,008511	-0,008511	-0,026586	6,643573
Proline	0,0013794	-0,70599	0,8261222	0,1355564	-0,201687	0,2806278
Putrescine	-0,015256	0,3183084	0,53552	-1,630777	-0,00394	-0,189001
Pyridine_ 2-hydroxy-	-0,684846	-0,807926	2,776916	-3,37904	1,840563	0,2624835
Pyroglutamic acid	0,0418386	2,318697	-2,695338	-1,495782	6,652765	-293,3044
Pyrophosphate	0,2186994	-1,132786	-0,010506	4,405611	-0,815873	0,6205068
Pyruvic acid_ 3-hydroxy-	-1,350655	1,037624	-1,067408	2,576241	0,6639711	-0,892523
Pyruvic acid	-0,597815	1,529382	-0,900832	0,2376296	-8,410924	7,777458
Rhamnose	-1,53004	-0,744237	0,3142999	2,311697	229,1542	-5,201877
Ribonic acid	0,75	-0,010947	-0,010947	-0,010947	0,75	-0,008681
Ribose_ 2-deoxy-	0	0	0	0	0	0
Ribose	0,0744157	0,0415863	-0,12107	-0,272415	2,053853	-0,842247
Ribose-5-phosphate	0	0	0	0	-0,000742	0,75
Ribulose	-0,564027	0,6626948	-1,94618	-1,489452	9,557563	-4,778345
Ribulose-5-phosphate	-1,190081	-3,825959	1,293986	1,335488	-2,576399	-3,104067
Serine	-0,587718	1,423748	-4,244702	11,4695	-0,560511	-0,494916
Sorbitol	0	0	0	0	0	0

Spermidine	-1,785594	0,4883509	0,0151897	8,385289	-17,61572	-0,325532
Succinic acid	0,2098245	0,6144703	-5,271265	2,501369	1,479675	-0,845473
Sucrose	-63,60122	-10,93212	11,23849	1,638278	0,2082135	0,5476474
Sucrose-6-phosphate	-0,653212	0,7074124	-3,883754	-0,009481	-0,392608	-2,681639
Threonic acid	-0,022743	0,75	-0,022743	-0,022743	0	0
Threonine	-0,462904	-1,498603	-31,90568	189,3123	-5,027353	-3,514321
Trehalose_a-a-	-3,534386	73,9021	-195,9356	14,25358	-181,3379	-1,211669
Triethanolamine	0,75	-0,002833	-0,002833	-0,002833	0	0
Tryptophan	0,067181	-4,234622	12,91668	6,95149	6,171301	0,0400047
Tyrosine	0,0774294	-0,4076	0,3361464	-0,054851	0,9119415	-1,87531
Uracil	-0,878712	0,3174241	1,351699	-0,768704	-0,984188	0,8357288
Urea	-0,023806	-0,023806	0,75	-0,023806	-0,004478	0,4357416
Uric acid	1,229951	-0,877475	-1,40244	0,9546744	-0,035445	-0,010021
Uridine 5'-monophosphate	0,3086619	-0,2498	-0,363516	0,5072317	-0,291961	-0,032494
Uridine 5'-monophosphate	0,3086619	-0,2498	-0,363516	0,5072317	-0,291961	-0,032494
Uridine	-1,261887	0,3699474	2,611074	-1,141252	-0,065544	0,4054645
Valine	-0,162006	-0,818809	6,540714	-3,614793	-0,150623	-7,863975
Xylose	0,5167596	-0,561737	-0,004429	0,225001	3,687578	-0,343183
